# Neurogenic locus notch homolog protein 1 (NOTCH 1) SNP informatics coupled with intrinsically disordered regions and post-translational modifications reveals the complex structural crosstalk of Lung Adenocarcinoma (LUAD)

**DOI:** 10.3389/fbinf.2025.1641521

**Published:** 2025-12-10

**Authors:** Pearl John, C. Sudandiradoss

**Affiliations:** Department of Biotechnology, School of Biosciences and Technology, Vellore Institute of Technology, Vellore, Tamilnadu, India

**Keywords:** lung adenocarcinoma, neurogenic locus notch homolog protein 1, deleterious SNP, intrinsically disordered region, post-translational modification, molecular dynamics simulation, epithelial–mesenchymal transition signaling

## Abstract

**Background:**

Lung adenocarcinoma (LUAD) is the predominant histological subtype of lung cancer, representing a major contributor to cancer mortality rate marked by a high frequency of mutations and intricate interactions between multiple signalling pathways.

**Objective:**

Here we explore the role of NOTCH1 associated Single nucleotide polymorphisms (SNPs) IDR and PTM in LUAD progression. Although the NOTCH1 expression is downregulated, it has been validated as an important prognostic marker because of its complex biological roles under specific conditions.

**Methods:**

With the aid of In silico tools we predicted and identified the deleterious SNPs. The Molecular Docking and dynamics simulations (MDS) were conducted to characterize these mutations.

**Results:**

A total of 43 deleterious SNPs were found in the sequential SNP analysis with 13 SNPs resulted deleterious and damaging effects. The stabilizing SNPs such as S1464I, A1705V and T1602I are found within the conserved and functional domains of NOTCH1. In addition, 1660–2555 sequence region of the PEST domain was recognized as an Intrinsically Disordered Region (IDR) with a score of above 0.5. Moreover, the presence of the two phosphodegrons (SCF_FBW7_1 at 2129–2136 and SCF_FBW7_2 at 2508–2515) along with the Post Translational Modification (PTM) such as o-linked glycosylation and Phosphothreonine within the IDR region, PEST and conserved domains suggest functional significance in LUAD progression.

**Conclusion:**

In conclusion our research highlights the potential regulatory role of identified SNPs, PTMs, and the functional domains of Notch1, particularly the PEST domain and IDR, in pathophysiology of LUAD particularly through the crosstalk of the EMT signalling.

## 1 Introduction

Lung cancer remains a leading cause of global mortality, accounting for 85% of cases (Matsuda and Machii, 2015), with lung adenocarcinoma (LUAD) being the most common histopathologic subtype among non-small-cell lung cancers (NSCLCs) (Tyczyński and Parkin, 2010; Li M. et al., 2024) Some of the prominent risk factors that play a part in the etiology of LUAD are lifestyle, genetic, and epigenetic factors (Ullah et al., 2024); metabolic activities; environmental and occupational exposure; and complex signaling pathways (Li et al., 2022). The key existing obstacles for screening and treating LUAD are a lack of an early detection system, tumor recurrence, and drug resistance. Thus, more ideas are needed for enhancing the prognosis of LUAD patients through novel molecular markers and prognostic models and by addressing the aforementioned risk factors. Among the mentioned existing risk factors, the metabolic reprogramming and the complex signaling pathways play a crucial role in promoting metastasis, resulting in metabolic heterogeneity in adenocarcinoma (Sellers et al., 2019; Soga, 2013). In LUAD, NOTCH1 facilitates tumor development by inducing epithelial–mesenchymal transition (EMT) through both direct modulation of transcription factors such as SLUG, an E-box-binding TF that drives the EMT signaling by repressing the epithelial and polarity genes, and indirect interplay with other EMT-associated pathways within the tumor microenvironment (John and Sudandiradoss, 2024a).

The Notch pathway is implicated in various malignancies, playing diverse roles through mutations, deletions, amplifications, and altered expression of its receptors. Until now, the functional impact of these roles remains undefined in tumor initiation, progression, and therapy (Rolle et al., 2021). Notch signaling is one of the most conserved pathways and plays a crucial role in intercellular communication, the fate of cells, cell differentiation, and the proliferation of tumor cells. Signaling in this pathway is the juxtacrine cellular signaling method, which governs cellular differentiation, apoptosis, growth, and the development of various organs. The pathway consists of five ligands, namely, JAG1, JAG2, DLL1, DLL3, and DLL4. The receptors involved in this pathway are known as the Notch receptors and are named Notch-1, Notch-2, Notch-3, and Notch-4, which are transmembrane proteins with extracellular and intracellular domains. The NOTCH1 receptor contains several functional domains, such as the PEST domain, NOD domain, and NOTCH domain, which play a crucial role in LUAD progression. Among the reported domains, the PEST (the domain that is rich in proline, glutamic acid, serine, and threonine) domain plays a crucial role in the degradation of the NICD, its stability, and the overall signaling pathway. In the PEST domain region, there is a short amino acid sequence that can be targeted for degradation using E3 ligases known as degrons. According to the existing reports, these regions, where degrons are present, could destabilize NOTCH1 and result in LUAD progression (Westhoff et al., 2009). The reported studies have proven the crucial role of NOTCH1 in lung cancer and NSCLC, and according to the clinical trials reports, 30% of primary human NSCLCs exhibited Notch signaling activation by either NOTCH1 overexpression or numb downregulation (Yuan et al., 2014; Westhoff et al., 2009). Thus, emerging studies indicate that the Notch pathway and its four receptors are involved in tumor biology in both oncogenic and tumor suppressor roles.

Owing to their efficacy and cost-effectiveness, *in silico* approaches offer greater insights than experimental studies in analyzing deleterious non-synonymous SNPs (nsSNPs) and their associated alterations (Kumar et al., 2023; Hossain et al., 2020). In tumor studies, it aids in emphasizing the key genes, their impact on pathway mechanisms, and their structure and function in tumor cells (Buljan et al., 2018; Mészáros et al., 2021). Our previous Notch-based network analysis in LUAD identified five genes, which include NOTCH1 as the TSG (tumor suppressor gene) and prognostic marker for LUAD progression through the crosstalk between the EMT and Notch signaling (John and Sudandiradoss, 2024a; John and Sudandiradoss, 2024b). Thus, changes in cellular metabolism and signaling pathways, such as EMT and Notch, and the crosstalk between these pathways and key genes are some of the main drivers of carcinogenesis, tumor development, and chemotherapeutic resistance. These are regarded as hallmarks of cancer. Hence, considering the aforementioned hallmarks, we utilize *in silico* approaches to highlight and analyze the functional variants of the NOTCH1 protein in LUAD progression, which might be useful for further research studies.

NOTCH 1 of 272.50 kDa subunits is a protein encoded by the NOTCH1 gene that comprises 2,555 amino acids. It is a key gene in the highly conserved Notch signaling pathway, which plays a vital role in cell development and cell fate determination. The key roles of NOTCH1 in the tumor cells are angiogenesis, differentiation, proliferation, and migration (Zhou et al., 2022). The frequently altered expression of NOTCH1 has been reported as oncogenic in cancers such as lung, T-ALL, colon, breast, ovarian, and hepatocellular cancer and gliomas and is often correlated with poor survival of patients (Zhou et al., 2022). The interacting partners, such as PSEN1 (Song et al., 2022), DLL4 (You et al., 2023), MAML1, MAML2 (Kim et al., 2020), JAG2 (Mandula et al., 2022), and JAG1 (He et al., 2023; Chang et al., 2016) of NOTCH1, also have a crucial role in LUAD progression; however, the underlying mechanism in LUAD progression is yet to be defined.

Intrinsically disordered regions (IDRs), single nucleotide polymorphisms (SNPs), and post-translational modifications (PTMs) and the changes in the functional domain have an impact on tumor development. However, it is still unclear whether the role of IDR mutation has an impact or not (Deng et al., 2017; Mészáros et al., 2021). The presence of post-translational modifications in the disordered regions, especially in the PEST domain of NOTCH1, highlights that readily accessible and flexible regions are more preferable for the modifications (van der Lee et al., 2014). The PTMs, deleterious SNPs, IDRs, and PTMs in the IDR regions, as critical regulators of cancer cells present in NOTCH1, may be involved in processes by which cancer cells revamp and lead to tumorigenesis (Li M. et al., 2024). Thus, in this study, we utilize *in silico* methods to analyze the variants in the *NOTCH1* gene, how the deleterious SNPs affect the function, and the effect of the IDRs and PTMs in *NOTCH1* gene stimulating LUAD progression. As of yet, there is no *in silico* approach of SNP analysis that has been reported in the *NOTCH1* gene in correlation with LUAD.

## 2 Methods

### 2.1 Collection and validation of datasets

The *NOTCH1* gene SNP information has been retrieved from the dbSNP of NCBI (https://www.ncbi.nlm.nih.gov/snp/) and the COSMIC (Catalog of Somatic Mutations in Cancer) (https://cancer.sanger.ac.uk/cosmic) mutational database. The sequence of the *NOTCH1* gene in FASTA format was obtained from the Universal Protein Database (UniProt ID: P46531). The structural and functional analysis of NOTCH1 has been performed in various *in silico* tools using the sequence obtained from the UniProt database. Gene expression analysis has been performed in the three datasets, namely, GSE75037 (a total of 166 samples, with 83 tumor and 83 normal samples), GSE32863 (a total of 116 samples, with 58 tumor and 58 normal samples), and GSE10072 (a total of 58 tumor samples, with 49 normal samples). The GEPIA (Gene Expression Profiling Interactive Analysis) (http://gepia.cancer-pku.cn) tool was used to analyze the expression profile of NOTCH1 in the different stages of LUAD and in normal and tumor samples. The tool also provides log2 and adjusted *p*-values to analyze the overall gene expression (overexpressed or underexpressed). The overall survival plots have been validated using the KM (Kaplan–Meier) plotter (https://kmplot.com/), in which survival is represented as Kaplan–Meier survival curves that provide the hazard ratio (HR) values and *p*-values (<0.05) with a 95% confidence interval (CI).

### 2.2 Characterization of the functional effects in nsSNPs of NOTCH1 using sequence- and structure-based prediction methods

#### 2.2.1 Sequence-based prediction tools

##### 2.2.1.1 SIFT: sorting intolerant from tolerant

The SIFT tool (https://sift.bii.a-star.edu.sg/www/SIFT_dbSNP.html) was used to rule out deleterious nsSNPs. SIFT is a sequence homology-based tool that foresees the impact of the SNPs as deleterious or tolerated (Ng and Henikoff, 2003). The rs IDs (SNP IDs) or the protein sequences are provided as the query in the tool, which validates the input sequence using the sequence alignment information to forecast the tolerated and deleterious effects for the query sequence with a SIFT prediction score.

##### 2.2.1.2 PolyPhen-2: Polymorphism Phenotyping v2

For the structural-level analysis to analyze the damaged SNPs, the *in silico* tool PolyPhen (http://genetics.bwh.harvard.edu/pph2/) was used. The tool incorporates structural and physicochemical properties that evaluate the potentially damaging effects on the structure and function of the query protein caused by the amino acid substitution. The FASTA sequence of the protein with the positional change and amino acid substitution was provided as the query to the tool. Based on several sequences and phylogenetic and structural features characterizing the substitution, the tool determines whether the alteration at the specified position is damaging or not, with the scores and their effects categorized as HUMDIV and HUMVAR.

##### 2.2.1.3 Predict SNP

The Predict SNP tool (https://loschmidt.chemi.muni.cz/predictsnp1/) is used to evaluate the functional variations in the protein sequence due to single-point variants. Predict SNP considers almost all mutations from both PMD-UNIPROT and MMP testing datasets to evaluate and provide a consensus prediction. The tool interfaces are currently accessible for six tools, namely, MAPP, SIFT, PhD-SNP, PolyPhen-1, PolyPhen-2, and SNAP2, to predict the functional effects of the variants. The input of the tool is the protein sequence along with the positions of the mutations. Alternatively, we can provide the list of mutations in a text format as the query. After providing all the desired mutations and specifications, the user can select tools to be used for the evaluation of the selected mutations. The tool provides output in a tabular form that contains the PredictSNP confidence score, with different colors to differentiate between the neutral or deleterious and disease-causing or damaging effect.

##### 2.2.1.4 SNPs&GO

SNPs&GO (https://snps-and-go.biocomp.unibo.it/snps-and-go/) is a server that predicts whether an SNP is disease causing or not by utilizing the corresponding protein functional annotation. The tool incorporates encoded data related to the protein sequence, evolutionary information, and functional annotation that are encoded in Gene Ontology terms. The UniProt ID, along with the mutational position and changes in the amino acid residues, was provided as the query, which outputs the impact of the amino acid position change as disease or neutral, along with a relative index score. The tool also provides Gene Ontology terms, including molecular function, biological process, and cellular components, as the output with the index score.

##### 2.2.1.5 PANTHER: protein analysis through evolutionary relationships

The tool PANTHER (https://www.pantherdb.org/tools/csnpScoreForm.jsp) was used to predict the functional consequence of an SNP on a protein. PANTHER is an extensive knowledge base that includes insights into the evolutionary and functional relationships of proteins and tools for analyzing the enormous amount of genomics data. The tool analyzes the functional impact based on the preservation time, which is calculated from the conservation of a given amino acid sequence. The input to the tool was provided as the protein sequence with amino acid substitutions, specifying the organism, and it predicts the functional effect as damaging or benign, with the relative index score as the output.

#### 2.2.2 Structure-based prediction tools

In this study, we identified all the deleterious nsSNPs of the *NOTCH1* gene influencing the structure and function through sequence-based analysis, and they were further subjected to stability analysis using structure-based tools. The structural stability and flexibility of proteins vary based on the energy differences (ΔΔ*G*) and the entropy (ΔΔS) values.

##### 2.2.2.1 I-Mutant 2.0

I-Mutant 2.0 is a support vector machine (SVM)-based server used to predict how single-point mutations could impact protein stability. The stability prediction is carried out using either the sequence or the structure with the stability values (ΔΔ*G*). The ProTherm (thermodynamic database for proteins and mutants) database was used for the cross-validation to ensure the validity of the predicted values. Approximately 77%–80% of the predictions from I-Mutant are based on the sequences or structure.

##### 2.2.2.2 DynaMut

DynaMut (https://biosig.lab.uq.edu.au/dynamut/) is used for anticipating the impact of mutations on protein conformation, flexibility, and stability. The stability of the protein structure was evaluated using normal mode approaches with graph-based signatures to predict stability through the free energy and entropy values. The tool generates approximately 2,297 mutations and executes a blind test to analyze the impact of the mutations on free folding energy. The vibrational entropy (ΔΔS) of the mutations was analyzed via the normal mode analysis. The query is provided as the PDB structure with the mutational changes specifying the position and yields the stability results as stabilizing or destabilizing, with the stability value (ΔΔG) and entropy value (ΔΔS).

### 2.3 Identification of the conserved domains and biophysical characters of NOTCH1

The ConSurf server is an *in silico* tool used to evaluate and analyze conserved regions in a protein molecule. HMMER, as a homology algorithm, and the Bayesian method were used in the tool for calculation, which estimated the evolutionary conservation of the amino acid positions. The query in the tool is the 3D structure of the protein, which outputs the conservation score (CS), ranging from 0 (least conserved) to 9 (highly conserved). The conservation scores are divided into three categories, namely, variable (CS: 1–4), mean score (CS: 5–6), and conserved (CS: 7–9). The biophysical attributes of NOTCH1 were also evaluated using the HOPE server. The data from UniProt, PDB, and Distributed Annotation System (DAS) are gathered on the server to analyze the functional impact of the mutation in the protein structure. The pathogenic effect of the structural representations, properties, and conservation analysis on the mutation in the structure is provided as the output of the tool.

### 2.4 Prediction of intrinsically disordered regions and post-translational modifications in NOTCH1

#### 2.4.1 MusiteDeep

The prediction and visualization of post-translational modification sites in the protein were evaluated using MusiteDeep. The tool uses protein sequences and selected PTMs as the input and uses the deep learning method to predict the PTM sites in the protein. The results obtained from the tool are represented as the post-translational modification letters on top of the amino acid residue where the modifications are present. In addition to the PTM prediction, the protein’s 3D structure was also obtained using a homology-based search. Although MusiteDeep is a deep-learning-based approach for predicting the PTM sites, its predictions are subject to certain limitations, such as the performance of the tools being dependent on the quality and diversity of the training data, which may produce biased results toward the well-known PTMs such as phosphorylation. Additionally, the tool predicts the outcome based only on the sequence context and does not incorporate structural or expression-related information, which can influence the PTM occurrence.

#### 2.4.2 IUPred3

The disordered and ordered region prediction in the protein sequence is evaluated using the IUPred web-tool. Here, the query in the tool is given as a protein sequence or UniProt ID/accession to predict the probability of each amino acid being in a disordered region. The tool evaluates the disordered region analysis in three options, namely, long disorder, short disorder, and structured domain, and it also provides an option for predicting context-dependent disordered regions in the protein sequence. The output of the tool contains a graphical representation with an IDR score in which the residues with scores above 0.5 are predicted to be disordered, while those with scores below 0.5 are considered ordered. The Pfam analysis in the tool provided the output regarding the number of functional domains present in the protein. The outcomes are cross-validated with the experimental data incorporated in the tool. ANCHOR2, a new tool incorporated in IUPred, predicts the regions of disorder-to-order transition upon binding to another protein. The disordered binding sites having a score above 0.5 are considered the context-dependent disordered region.

### 2.5 Decoding degrons: protein degron prediction and mapping using DEGRONOPEDIA

Protein degron prediction was carried out using the tool DEGRONOPEDIA (https://degronopedia.com/), which is an open-source tool that identifies degrons and associates them with nearby residues that are potential sites for ubiquitination and disordered regions that can serve as initiation points for protein unfolding. In addition to ruling out the degrons in the protein, the server also provides evolutionary insights into degron conservation and their scores and predicts the post-translational modifications and mutations that may affect degron availability. The tool uses the input as the protein UniProt ID or the sequence in FASTA format and the structure of the protein. Using the above-mentioned inputs, the server provides the output as the number of degrons present in the protein with the experimental and predicted proteolytic sites.

### 2.6 Molecular docking analysis of NOTCH1 and its interactors using HADDOCK

The protein–protein docking approach was carried out using the tool HADDOCK 2.4 (https://wenmr.science.uu.nl/), in which the interaction analysis is based on biochemical or biophysical interaction data. The structures of proteins (interactors of the query protein) were provided as the input with the active residue position and chain specifications. The information provided was utilized to carry out docking. The docked complexes are categorized based on their intermolecular energy, which is composed of electrostatic, van der Waals, and AIR energy. Z-scores, docking scores, graphical representation of the energies, and PDB structures of the docked complexes are the major outcomes of HADDOCK. In HADDOCK, the most accountable cluster is the top cluster, having the maximum Z-score. The Z-score indicates how many standard deviations away from the average this cluster is located in terms of the score (the more negative the Z-score, the more reliable the obtained structure).

### 2.7 Molecular dynamics simulation

The MD simulation was performed by the GROMACS 2023.1 version dynamics program to expose the changes at the atomic level for native and mutant NOTCH1 and its interactor complexes. Prior to applying the simulation, the structures of NOTCH1 and mutants were cleaned, and the OPLS/AA force field was applied in the simulation. Using the Genion tool, a cubic box (with a side of 1.0 nm) was created by adding and covering the protein with H atoms; the box was filled with the SPC216 water model, and the charge of each system was neutralized by adding Na^+^ or Cl^−^ ions, accordingly. Subsequently, using the G energy tool, the steepest descent minimization algorithm was used to minimize energy until it reached 1,000 kJ mol^−1^ nm^−1^. Temperature and pressure optimization were carried out by restraining the heavy atoms of the protein with the application of force. Then, a production MD run of 100 ns timescale was started for each system. To determine the RMSD, RMSF, radius of gyration, and potential energy, the obtained trajectory files were evaluated. The docked complexes were analyzed using the CABS-flex tool, which requires the input file as PDB files, and dynamic properties of the molecule were evaluated using the rigid motion of the protein complex to determine flexibility. The trajectory files were obtained as the output and were further evaluated to obtain RMSF graphs.

## 3 Results

### 3.1 Validation of NOTCH1 and the collection of nsSNPs

**FIGURE 1:**
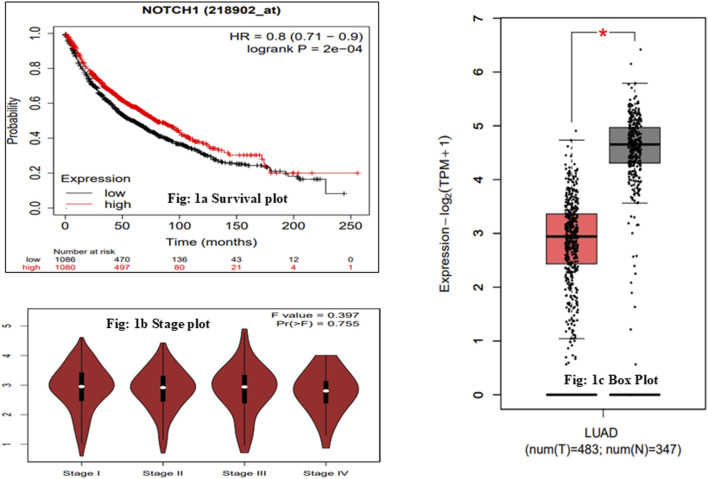
Expression analysis of NOTCH1 in LUAD. **(a)** Overall survival analysis of NOTCH1 in LUAD based on the hazard ratio (HR). HR > 1 suggests an increased risk, and a hazard ratio below 1 suggests a smaller risk. **(b)** Expression of NOTCH1 in advanced clinical stages of LUAD. The gene expressions of the hub genes in advanced clinical stages are shown. The expression is represented in terms of log2 (TPM +1). **(c)** Box plot of NOTCH1 in LUAD, in which red indicates the tumor samples and gray indicates the normal samples. The box plots represent the median values of hub genes in the tumor stage compared to those in normal condition.

To validate the expression of NOTCH1 in LUAD patients, we carried out an expression analysis and survival analysis of NOTCH1 across all four tumor stages with log2 (FC) values. The obtained log2 (FC) value of NOTCH1 is −1.71, which is graphically represented in box and stage plots, and the overall survival analysis of NOTCH1 using the KM-plotter resulted in an HR value of 0.8, 95% CI = 0.71–0.9, and p = 2e-04 (Figures 1a–c). The results indicate that NOTCH1 expression is significantly associated with improved overall survival, with an HR less than 1, suggesting a protective effect in the analyzed cohort. Thus, from the expression analysis, NOTCH1 has been validated as playing an essential role in LUAD patients. Furthermore, we collected 30,288 nsSNPs from the National Center for Biotechnology Information (NCBI), dbSNP, and COSMIC databases. Following the process of filtration and duplicate elimination, 4,937 SNPs were gathered for further analysis.

### 3.2 Elucidation of deleterious mutation based on the structural and sequence-based prediction

**FIGURE 2:**
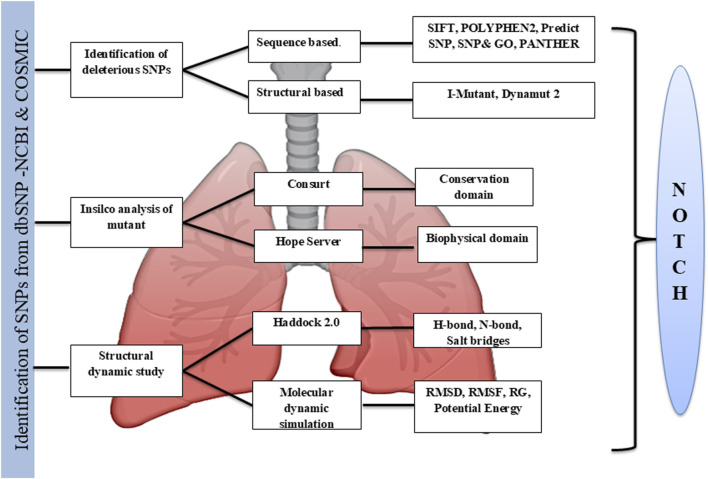
Stepwise screening of deleterious nsSNPs in the *NOTCH1* gene using multiple computational prediction tools. From a total of 4,937 missense SNPs, variants with SIFT scores ≤0.05 were identified, yielding 43 as potentially deleterious SNPs. These were further filtered using PolyPhen-2 (retaining variants classified as possibly/probably damaging; score >0.85), SNPs&GO (>0.5), PANTHER (subPSEC-3.0; probability ≥0.5), and PredictSNP (deleterious confidence >60%). This multi-tool, cross-validation workflow minimized prediction bias and ensured high-confidence identification of deleterious nsSNPs for downstream analysis, resulting in 13 SNPs.

**FIGURE 3:**
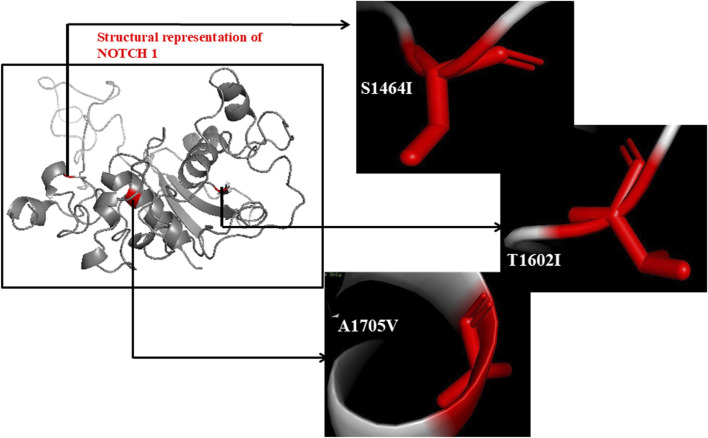
Structural representation of the NOTCH1 protein. The red color highlighted in the structure indicates identified deleterious disease-causing mutants such as S14641, T16021, and A1705V with a stability score MG value of 0.62, 0.13, and 0.56, respectively.

The *NOTCH1* gene’s non-synonymous SNPs were gathered from multiple databases, and the most harmful and deleterious SNPs were assessed using a variety of prediction techniques that used both the sequence and the structure (Figure 2). A total of 4,937 missense SNPs were collected from the dbSNP and COSMIC databases, which were analyzed in the SIFT analysis to investigate the deleterious effect, which provided a total of 43 deleterious mutations based on the predicted score (≤0.05) (Supplementary Table S1). Apart from that, the 43 identified deleterious mutations were subjected to the PolyPhen tool (PolyPhen score >0.85) to evaluate the damaging effects of the SNP. The PredictSNP tool was used as a consensus classifier for identifying disease-causing and deleterious effects, predicting SNPs as deleterious and disease causing with an accuracy of >60%. Furthermore, the SNPs&GO tool predicts deleterious and disease-causing effects with a probability score of >0.5, and the scores ≤0.5 were considered neutral. The PANTHER tool uses the evolutionary conservation scores to predict the damaging effect with a subPSEC (substitution position-specific evolutionary conservation) score of ≤ −3.0 and a probability of deleterious effect of ≥0.5. This step-wise multi-tool cross-validation approach ensured methodological rigor by integrating predictive algorithms based on different theoretical principles, such as sequence biology, evolutionary conservation, structural modeling, and functional annotation, which reduces the tool-specific bias and enhances the robustness of deleterious SNP identification for subsequent structural and functional analyses. Moreover, to obtain accurate predictions, we moved forward the 13 identified deleterious mutations with tools such as PredictSNP, SNPs&GO, and PANTHER to screen the disease-causing mutations provided in Table 1. The 13 identified SNPs with deleterious and damaging effects were further subjected to structure prediction tools to determine the stability profile of SNPs. We performed the stability analysis mainly through two ways, namely, sequence-based (I-Mutant 2.0) and structure-based (DynaMut) analyses, in the 13 identified deleterious SNPs, which resulted in three SNPs, namely, S1464I, A1705V, and T1602I, as the stabilizing ones with an *ΔΔ*G value of 0.62 for S1464I, 0.13 for A1705V, and 0.56 kcal/mol for T1602I (Figure 3) (Table 1).

**TABLE 1:** Identification and prediction of deleterious and damaging SNPs of NOTCH1 with the stability profile leveraging *in silico* approaches.

SNP	Amino acid position and substitution	SIFT prediction with score	PolyPhen-2 prediction with score	PredictSNP prediction with score	SNPs&GO prediction with score	PANTHER prediction with score	I-Mutant 2.0 prediction with RI value and DDG value Kcal/mol	DDG value of DynaMut-2 Kcal/mol
rs371333249	G275S	Deleterious-0.009	Probably damaging = 1.000	Disease	Disease RI = 2	Probably damaging = 0.74	Decrease RI = 8 −1.10	Destabilizing −0.63
rs201236538	A348P	Deleterious-0.003	Possibly damaging = 0.880	Disease	Disease RI = 1	Probably damaging = 0.57	Decrease RI = 1 −1.08	Destabilizing −0.02
rs200520088	T349P	Deleterious-0.003	Probably damaging = 1.000	Disease	Disease RI = 9	Probably damaging = 0.85	Decrease RI = 1 −0.63	Stabilizing 0.16
rs376055493	T559M	Deleterious-0.011	Probably damaging = 0.927	Disease	Disease RI = 7	Possibly damaging = 0.5	Decrease RI = 6 −0.46	Stabilizing 0.2
rs374434131	G766V	Deleterious-0.001	Probably damaging = 1.000	Disease	Disease RI = 9	Probably damaging = 0.85	Decrease RI = 7 −1.42	Destabilizing −1.48
rs371050668	P877L	Deleterious-0.001	Probably damaging = 1.000	Disease	Disease RI = 9	Probably damaging = 0.85	Decrease RI = 8 −0.43	Destabilizing −0.77
rs375190395	S1168F	Deleterious-0.042	Probably damaging = 0.962	Disease	Disease RI = 7	Possibly damaging = 0.5	Decrease RI = 5 −0.07	Destabilizing −0.32
-	S1464I	Deleterious-0.00	Possibly damaging- 0.494	Disease	Disease RI = 9 dbNSFP: 0.9324	Possibly damaging = 0.5	Increase RI = 4 0.62	Stabilizing 1.22
-	T1602I	Deleterious-0.04	Probably damaging = 0.999	Disease	Disease RI = 9 dbNSFP: −0.9870	Probably damaging = 0.74	Increase RI = 5 0.56	Stabilizing 0.15
-	A1705V	Deleterious-0.01	Probably damaging = 0.998	Disease	Disease RI = 9 dbNSFP: 0.9490	Probably damaging = 0.74	Increase RI = 3 0.13	Stabilizing 0.93
rs373806373	R2087W	Deleterious-0.001	Probably damaging = 0.994	Disease	Disease RI = 4	Probably damaging = 0.85	Decrease RI = 7 −1.08	Stabilizing 0.4
rs375969725	R2120C	Deleterious-0.002	Probably damaging = 0.996	Disease	Disease RI = 7	Probably damaging = 0.57	Decrease RI = 7 −2.42	Stabilizing 0.79
rs374453977	R2313W	Deleterious-0.006	Probably damaging = 0.999	Disease	Disease RI = 8	Probably damaging = 0.74	Decrease RI = 8 −1.86	Destabilizing −0.52

### 3.3 Elucidation of the intrinsically disordered region and post-translational modifications in NOTCH1

The human sequence of NOTCH1 was analyzed for the prediction of IDRs and PTMs using MusiteDeep and IUPred 3. We identified approximately 10 types of post-translational modifications, such as hydroxyproline, O-linked glycosylation, phosphothreonine, phosphoserine, N-linked glycosylation, phosphotyrosine, pyrrolidine–carboxylic acid, SUMOylation, ubiquitination, N6-acetyllysine, methyllysisne, and methylariginine, in the NOTCH1 sequence provided in Supplementary Table S2. The sequence of NOTCH1 shows the IDR regions at the residue positions after 1,600 in parts. The predicted IDR regions in the sequence are 1,660–1,666, 1,723–1,725, 1,781–1,821, 1,833–1,877, 1,889–1,910, 1,923–1,936, 1,959–1,966, 2,053–2,068, 2,094–2,103, 2,124–2,195, 2,202–2,250, 2,259–2,284, 2,321–2,353, and 2,366–2,555. These regions depict the peaks in the IDR graph, showing a disordered character with an IDR score of 0.5 and above. The context-dependent disordered binding sites in the sequences were identified through the ANCHOR2 analysis. The prediction plot is in different colors, in which the disordered prediction profile is red and the disordered binding sites are blue. Our predicted results show the same increase in the peak of the IDR-predicted region, indicating that these regions undergo a disorder-to-order transition upon binding to another protein.

**FIGURE 4:**
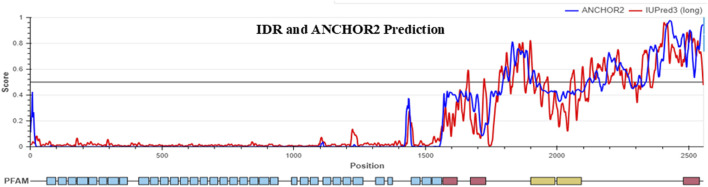
IDR prediction and disorder-to-order transition prediction in NOTCH1 using IUPred3. The red line depicts the IDR graph having the peaks at the position from 1,600 to 2,555 in parts. The blue line depicts the ANCHOR-2 disorder prediction plot of the NOTCH1 sequence showing a slight increase in the peak of same IDR predicted regions. The Pfam analysis from IUPred3 shows two functional domains in NOTCH1 sequences, such as the EGF domain of residues from 66–1,344, the Notch domain of residues 1,446–1,562, the NOD family domain of residues 1,566–1729, the ANK_2 repeats of residues 1,990–2,092, the DUF3454 of residues 2,479–2,540, and the PEST domain of residues 2,478–2,541.

The IDR and ANCHOR 2 predictions resulted in the identification of the sequence family information in which our results ruled out the functional domains such as the EGF domain of residues from 66–1,344, the Notch domain of residues 1,446–1,562, the NOD family domain of residues 1,566–1,729, the ANK_2 repeats of residues 1,990–2,092, DUF3454 of residues 2,479–2,540, the PEST domain of residues 2,478–2,541, and phosphorylation-dependent degradation site 2,508–2,515 (see Table 2 and Figure 4).

**TABLE 2:** Prediction of intrinsically disordered regions in NOTCH1 sequence using IUPred 3. The table displays the anticipated IDR areas in the sequence along with their residue number and the position for regions with scores greater than 0.5.

Residue position	Amino acid residue	Intrinsically disordered region score
1,660, 1,661, 1,662, 1,663, 1,664, 1,665, 1,666	G, R, R, R, R, E, L	0.5231, 0.5591, 0.579, 0.5923, 0.5939, 0.5793, 0.5395
1,723, 1,724, 1,725	S, E, T	0.5071, 0.5292, 0.5241
1,781, 1,782, 1,783, 1,784, 1,785, 1,786, 1,787, 1,788, 1,789, 1,790,1,791, 1,792, 1,793, 1,794,1,795,1,796, 1,797, 1,798, 1,799, 1,800, 1,801, 1,802, 1,803, 1,804, 1,805, 1,806, 1,807, 1,808, 1,809, 1,810, 1,811, 1,812, 1,813, 1,814, 1,815, 1,816, 1,817, 1,818, 1,819, 1,820, 1,821	K, K, R, R, E, P, L, G, E, D, S, V, G, L, K, P, L, K, N, A, S, D, G, A, L, M, D, D, N, Q, N, E, W, G, D, E, D, L, E, T, K	0.5237, 0.5517, 0.5749, 0.5902, 0.5963, 0.5916, 0.5753, 0.5784, 0.5968, 0.6246, 0.6243, 0.607, 0.589, 0.574, 0.5575, 0.5526, 0.543, 0.533, 0.5266, 0.5246, 0.5371,0.5578, 0.5833, 0.6052, 0.6144, 0.6359, 0.6614, 0.7031, 0.7275, 0.7299, 0.7075, 0.6777, 0.6432, 0.6228, 0.6131, 0.5994
1,817, 1,818, 1,819, 1,820, 1,821, 1,833, 1,834, 1,835, 1,836, 1,837, 1,838, 1,839, 1,840, 1,841,1,842,1,843, 1,844, 1,845, 1,846,1,847, 1,848, 1,849, 1,850, 1,851, 1,852, 1,853, 1,854, 1,855,1,856, 1,857, 1,858, 1,859, 1,860, 1,861, 1,862, 1,863, 1,864, 1,865, 1,866, 1,867, 1,868, 1,869, 1,870, 1,871, 1,872, 1,873, 1,874, 1,875, 1,876, 1,877	D, L, E, T, K, D, L, D, D, Q, T, D, H, R, Q, W, T, Q, Q, H, L, D, A, A, D, L, R, M, S, A, M, A, P, T, P, P, Q, G, E, V, D, A, D, C, M, D, V, N, V, R	0.5806, 0.5622, 0.5461, 0.5289, 0.5171, 0.5095, 0.5387, 0.5511, 0.5677, 0.5917, 0.6113, 0.6315, 0.6405, 0.646, 0.6515, 0.645, 0.6507, 0.6497, 0.6553, 0.6334, 0.5995, 0.5738, 0.5755, 0.5986, 0.6319, 0.6539, 0.6911, 0.7373, 0.7737, 0.7957, 0.7987, 0.7928, 0.7843, 0.7725, 0.761, 0.7482, 0.7443, 0.7461, 0.7579, 0.7632, 0.7807, 0.7972, 0.8094, 0.8013, 0.7768, 0.7413, 0.6996, 0.6434, 0.5745, 0.5103
1,889, 1,890, 1,891, 1,892, 1,893, 1,894, 1,895, 1,896, 1,897, 1,898, 1,899, 1,900, 1,901, 1,902, 1,903, 1,904, 1,905, 1,906, 1,907, 1,908, 1,909, 1,910	S, C, S, G, G, G, L, E, T, G, N, S, E, E, E, E, D, A, P, A, V, I	0.5062, 0.513, 0.5225, 0.5444, 0.5844, 0.6242, 0.6589, 0.6991, 0.7451, 0.7956, 0.8222, 0.8217, 0.8071, 0.777, 0.7511, 0.7097, 0.6617, 0.6207, 0.5871, 0.5569, 0.5282, 0.5008
1,923, 1,924, 1,925, 1,926, 1,927, 1,928, 1,929, 1,930, 1,931, 1,932, 1,933, 1,934, 1,935, 1,936	Q, T, D, R, T, G, E, T, A, L, H, L, A, A	0.5051, 0.5223, 0.5291, 0.532, 0.5384, 0.5423, 0.5495, 0.5592, 0.5711, 0.5638, 0.5552, 0.5423, 0.5298, 0.5013
1,959, 1,960, 1,961, 1,962, 1,963, 1,964, 1,965, 1,966	N, M, G, R, T, P, L, H	0.5089, 0.5152, 0.5183, 0.5229, 0.5309, 0.5434, 0.5399, 0.5249
2,053, 2,054, 2,055, 2,056, 2,057, 2,058, 2,059, 2,060, 2,061, 2,062, 2,063, 2,064, 2,065, 2,066, 2,067, 2,068	N, K, D, M, Q, N, N, R, E, E, T, P, L, F, L, A	0.5136, 0.521, 0.533, 0.5272, 0.5347, 0.5461, 0.5609, 0.5674, 0.5717, 0.581, 0.5878, 0.5973, 0.5874, 0.5598, 0.5291, 0.5043
2,094, 2,095, 2,096, 2,097, 2,098, 2,099, 2,100, 2,101, 2,102, 2,103	D, R, L, P, R, D, I, A, Q, E	0.5181, 0.5583, 0.5905, 0.6164, 0.6219, 0.6184, 0.5894, 0.5587, 0.5306, 0.5009
2,124, 2,125, 2,126, 2,127, 2,128, 2,129, 2,130, 2,131, 2,132, 2,133, 2,134, 2,135, 2,136, 2,137, 2,138, 2,139, 2,140, 2,141, 2,142, 2,143, 2,144, 2,145, 2,146, 2,147, 2,148, 2,149, 2,150, 2,151, 2,152, 2,153, 2,154, 2,155, 2,156, 2,157, 2,158, 2,159, 2,160, 2,161, 2,162, 2,163, 2,164, 2,165, 2,166, 2,167, 2,168, 2,169, 2,170, 2,171, 2,172, 2,173, 2,174, 2,175, 2,176, 2,177, 2,178, 2,179, 2,180, 2,181, 2,182, 2,183, 2,184, 2,185, 2,186, 2,187, 2,188, 2,189, 2,190, 2,191, 2,192, 2,193, 2,194, 2,195	L, H, G, A, P, L, G, G, T, P, T, L, S, P, P, L, C, S, P, N, G, Y, L, G, S, L, K, P, G, V, Q, G, K, K, V, R, K, P, S, S, K, G, L, A, C, G, S, K, E, A, K, D, L, K, A, R, R, K, K, S, Q, D, G, K, G, C, L, L, D, S, S, G	0.5006, 0.5449, 0.5872, 0.6194, 0.642, 0.6585, 0.6796, 0.6841, 0.6883, 0.6888, 0.6724, 0.6312,0.5993, 0.5845, 0.5728, 0.5563, 0.5343, 0.5278, 0.5385, 0.5517, 0.5495, 0.5369, 0.5213, 0.5179, 0.5229, 0.5292, 0.5432, 0.5685, 0.598, 0.6138, 0.6244, 0.6322, 0.6463, 0.6416, 0.6186, 0.6073, 0.6109, 0.6313, 0.636, 0.6323, 0.6238, 0.6188, 0.6176, 0.6141, 0.5975, 0.5946, 0.5952, 0.5897, 0.5828, 0.5862, 0.613, 0.6368, 0.6558, 0.6726, 0.6801, 0.6918, 0.6916, 0.6854, 0.6576, 0.6328, 0.6112, 0.5898, 0.5832, 0.5896, 0.5983, 0.6008, 0.5903, 0.5691, 0.5597, 0.5515, 0.5443, 0.5204
2,202, 2,203, 2,204, 2,205, 2,206, 2,207, 2,208, 2,209, 2,210, 2,211, 2,212, 2,213, 2,214, 2,215, 2,216, 2,217, 2,218, 2,219, 2,220, 2,221, 2,222, 2,223, 2,224, 2,225, 2,226, 2,227, 2,228, 2,229, 2,230, 2,231, 2,232, 2,233, 2,234, 2,235, 2,236, 2,237, 2,238, 2,239, 2,240, 2,241, 2,242, 2,243, 2,244, 2,245, 2,246, 2,247, 2,248, 2,249, 2,250	S, L, E, S, P, H, G, Y, L, S, D, V, A, S, P, P, L, L, P, S, P, F, Q, Q, S, P, S, V, P, L, N, H, L, P, G, M, P, D, T, H, L, G, I, G, H, L, N, V, A	0.5006, 0.5093, 0.521, 0.5227, 0.5393, 0.5492, 0.5518, 0.5459, 0.5476, 0.554, 0.5732, 0.5687, 0.5614, 0.5547, 0.5549, 0.5671, 0.5728, 0.5718, 0.5854, 0.6136, 0.6353, 0.6397, 0.6519, 0.6636, 0.6811, 0.6955, 0.7057, 0.7109, 0.7324, 0.7428, 0.7538, 0.7488, 0.7409, 0.7235, 0.6872, 0.641, 0.5952, 0.5693, 0.5565, 0.5506, 0.5518, 0.5624, 0.5783, 0.593, 0.5902, 0.578, 0.5513, 0.5245, 0.5069
2,259, 2,260, 2,261, 2,262, 2,263, 2,264, 2,265, 2,266, 2,267, 2,268, 2,269, 2,270, 2,271, 2,272, 2,273, 2,274, 2,275, 2,276, 2,277, 2,278, 2,279, 2,280, 2,281, 2,282, 2,283, 2,284	G, G, G, G, R, L, A, F, E, T, G, P, P, R, L, S, H, L, P, V, A, S, G, T, S, T	0.5031, 0.5307, 0.5608, 0.5833, 0.6015, 0.6019, 0.5958, 0.5719, 0.5531, 0.5385, 0.5379, 0.5428, 0.5532, 0.5639, 0.565, 0.5693, 0.5766, 0.5862, 0.6021, 0.6029, 0.6147, 0.6092, 0.6047, 0.5907, 0.5646, 0.5324
2,321, 2,322, 2,323, 2,324, 2,325, 2,326, 2,327, 2,328, 2,329, 2,330, 2,331, 2,332, 2,333, 2,334, 2,335, 2,336, 2,337, 2,338, 2,339, 2,340, 2,341, 2,342, 2,343, 2,344, 2,345, 2,346, 2,347, 2,348, 2,349, 2,350, 2,351, 2,352, 2,353	N, Q, Y, N, P, L, R, G, S, V, A, P, G, P, L, S, T, Q, A, P, S, L, Q, H, G, M, V, G, P, L, H, S, S	0.5314, 0.5755, 0.6016, 0.6289, 0.666, 0.6947, 0.71, 0.7117, 0.7166, 0.7111, 0.7218, 0.7312, 0.7428, 0.7488, 0.7432, 0.7316, 0.7254, 0.7332, 0.7412, 0.7492, 0.7384, 0.7267, 0.7179, 0.7141, 0.6993, 0.6695, 0.6325, 0.6024, 0.5805, 0.5543, 0.5434, 0.5267, 0.5105
2,366, 2,367, 2,368, 2,369, 2,370, 2,371, 2,372, 2,373, 2,374, 2,375, 2,376, 2,377, 2,378, 2,379, 2,380, 2,381, 2,382, 2,383, 2,384, 2,385, 2,386, 2,387, 2,388, 2,389, 2,390, 2,391, 2,392, 2,393, 2,394, 2,395, 2,396, 2,397, 2,398, 2,399, 2,400, 2,401, 2,402, 2,403, 2,404, 2,405, 2,406, 2,407, 2,408, 2,409, 2,410, 2,411, 2,412, 2,413, 2,414, 2,415, 2,416, 2417,2418, 2,419, 2,420, 2,421, 2,422, 2,423, 2,424, 2,425, 2,426, 2,427, 2,428, 2,429, 2,430, 2,431, 2,432, 2,433, 2,434, 2,435, 2,436, 2,437, 2,438, 2,439, 2,440, 2,441, 2,442, 2,443, 2,444, 2,445, 2,446, 2,447, 2,448, 2,449, 2,450, 2,451, 2,452, 2,453, 2,454, 2,455, 2,456, 2,457, 2,458, 2,459, 2,460, 2,461, 2,462, 2,463, 2,464, 2,465, 2,466, 2,467, 2,468, 2,469, 2,470, 2,471, 2,472, 2,473, 2,474, 2,475, 2,476, 2,477, 2,478, 2,479, 2,480, 2,481, 2,482, 2,483, 2,484, 2,485, 2,486, 2,487, 2,488, 2,489, 2,490, 2,491, 2,492, 2,493, 2,494, 2,495, 2,496, 2,497, 2,498, 2,499, 2,500, 2,501, 2,502, 2,503, 2,504, 2,505, 2,506, 2,507, 2,508, 2,509, 2,510, 2,511, 2,512, 2,513, 2,514, 2,515, 2,516, 2,517, 2,518, 2,519, 2,520, 2,521, 2,522, 2,523, 2,524, 2,525, 2,526, 2,527, 2,528, 2,529, 2,530, 2,531, 2,532, 2,533, 2,534, 2,535, 2,536, 2,537, 2,538, 2539,2540, 2,541, 2,542, 2,543, 2,544, 2,545, 2,546, 2,547, 2,548, 2,549, 2,550, 2,551, 2,552, 2,553, 2,554, 2,555	Q, G, L, P, S, T, R, L, A, T, Q, P, H, L, V, Q, T, Q, Q, V, Q, P, Q, N, L, Q, M, Q, Q, G, L, P, S, T, R, L, A, T, Q, P, H, L, V, Q, T, Q, Q, V, Q, P, Q, N, L, Q, M, Q, A, D, V, Q, P, L, G, P, S, S, L, A, V, H, T, I, L, P, Q, E, S, P, A, L, P, T, S, L, P, S, S, L, V, P, P, V, T, A, A, Q, F, L, T, P, P, S, Q, H, S, Y, S, S, P, V, D, N, T, P, S, H, Q, L, Q, V, P, E, H, P, F, L, T, P, S, P, E, S, P, D, Q, W, S, S, S, S, P, H, S, N, V, S, D, W, S, E, G, V, S, S, P, P, T, S, M, Q, S, Q, I, A, R, I, P, E, A, F, K	0.5125, 0.5246, 0.5476, 0.5657, 0.5751, 0.5923, 0.5904, 0.5918, 0.6014, 0.6279, 0.6648, 0.6991, 0.72, 0.7237, 0.7294, 0.7376, 0.7444, 0.7501, 0.7403, 0.7305, 0.7405, 0.7506, 0.766, 0.7771, 0.7673, 0.7693, 0.7728, 0.7849, 0.7965, 0.8006, 0.795, 0.781, 0.772, 0.7756, 0.7928, 0.8149, 0.8321, 0.8583, 0.8884, 0.9224, 0.9521, 0.9639, 0.9593, 0.954, 0.9491, 0.9484, 0.9434, 0.9399, 0.9361 0.9308, 0.9302, 0.94, 0.953, 0.959, 0.9521, 0.9287, 0.8999, 0.856, 0.8078, 0.7479, 0.6981, 0.665, 0.644, 0.6449, 0.6635, 0.6904, 0.7232, 0.7395, 0.7522, 0.7639, 0.7837, 0.7945, 0.7853, 0.7708, 0.771, 0.7675, 0.7744, 0.7674, 0.766, 0.7579, 0.7457, 0.7397, 0.7316, 0.7278, 0.7177, 0.7031, 0.6931, 0.6877, 0.6998, 0.7058, 0.7082, 0.6988, 0.6828, 0.6747, 0.6685, 0.6562, 0.6542, 0.6475, 0.6593, 0.6863, 0.7194, 0.7533, 0.7532, 0.7436, 0.7232, 0.6972, 0.6556, 0.626, 0.6198, 0.624, 0.6235, 0.6395, 0.6558, 0.6714, 0.6786, 0.6682, 0.6578, 0.6532, 0.6647, 0.6897, 0.707, 0.7343, 0.7667, 0.7998, 0.8142, 0.8268, 0.8425, 0.8736, 0.8863, 0.8844, 0.8728, 0.8646, 0.8453, 0.8275, 0.8151, 0.808, 0.8072, 0.8219, 0.8384, 0.8636, 0.8807, 0.8949, 0.8898, 0.8656, 0.8419, 0.8262, 0.8208, 0.8157, 0.8048, 0.806, 0.8131, 0.8248, 0.8329, 0.8412, 0.8381, 0.8361, 0.8299, 0.8285, 0.8087, 0.787, 0.7601, 0.734, 0.7148, 0.6996, 0.715, 0.7402, 0.7604, 0.7873, 0.8094, 0.8224, 0.8058, 0.7858, 0.7613, 0.7456, 0.7329, 0.7308, 0.7234, 0.7199, 0.7261, 0.7336, 0.737, 0.7235, 0.7087, 0.6886, 0.6635, 0.6344, 0.6025, 0.569, 0.5358, 0.5046

### 3.4 Functional annotation of degron landscape in NOTCH1

The UniProt ID of NOTCH1 was analyzed in DEGRONOPEDIA for the prediction of degrons with mutational characteristics, conservation scores, post-translational modifications, and interacting E3 ubiquitin ligases. We identified a total of 40 degrons in NOTCH1, with 25 degrons in the experimental proteolytic sites and 6,504 in the predicted proteolytic sites. Among the 40 degrons, the two degrons SCF_FBW7_1 at the position of 2,129–2,136 and SCF_FBW7_2 at the position of 2,508–2,515 are ones that are experimentally validated and known as phosphodegrons because of the presence of the phosphorylation PTM modification. The exact motifs of these phosphodegrons are LGGTPTLS and PFLTPSPE. The experimentally validated phosphodegron SCF_FBW7_2 is present in one of the functional domains known as the PEST domain of NOTCH1. Both the phosphodegrons are present in an IDR-predicted region with a score of 0.56 and 0.83. The pictorial representation of the protein sequence highlights the degrons and lysine residues (light blue). The figure also depicts structural features such as coils, buried residues, missense mutations, and various post-translational modifications (Supplementary Figure S1).

### 3.5 Conservation and biophysical analysis of NOTCH1

The conserved domains in NOTCH1 were examined using the ConSurf analysis algorithm to investigate nsSNPs S1464I, A1705V, and T1602I in a more comprehensive manner. The conservation analysis focused on the amino acid residues necessary for protein stability and how biomolecular interactions affect mutations through evolution. The evolutionary characteristics can potentially be useful in comprehending pathogenic mutations that impact human health. In the ConSurf tool, the evolutionary conservation of each amino acid residue was assessed from variable to highly conserved. Presumptive residues crucial for the structural stability and functional activity of NOTCH1 are identified by the conservation score. The amino acid residues of NOTCH1 are represented in different colors based on their conservation grade. Serine at position 1,464, threonine at position 1,602, and alanine at position 1,705 were present in the highly conserved domain of NOTCH1 with conservation scores of 8, 9, and 9, respectively. The conservation grade in the NOTCH1 sequence is given by a color-coding bar with scores ranging from variable to conserved (0–9). The region that scores the conservation grade of 0 to 4 belongs to the variable section (blue), and the scores from 5 to 6 correspond to the average conservation grade. The regions that score a conservation grade of 7 to 9 belong to the highly conserved region (pink). The residues S1464I, A1705V, and T1602I are positioned in the NOTCH, NOD, and NODP domains, resulting in a conservation score of 7–9, respectively (Supplementary Figure S2). The biophysical characteristics analysis of NOTCH1 and its mutants S1464I, A1705V, and T1602I was carried out using the HOPE server, which predicted them as pathogenic SNPs in the conserved domain. The mutant S1464I shows a neutral charge, while the wild-type shows a negative charge. The mutant residue A1705V is smaller than the wild-type, having a positive charge. Moreover, in the hydrophobicity aspect, the mutant A1705V is more hydrophobic with a neutral charge. The mutant T1602I is bigger in size and more hydrophobic than the wild-type residue.

As the positions of these residues are on the surface of the protein, the mutation will cause alterations in the interactions with other proteins and other parts of the protein. The SNP S1464I falls in the NOTCH domain (NICD domain), and the other two SNPs T1602I and A1705V fall in the NOD domain and NODP domain, which are functional and conserved regions where structural changes can disturb the domain and impair its function. The mutants were evaluated in the dbNSFP to measure pathogenicity, and it can range from 0.0 to 1.0. A higher score indicates greater pathogenicity. The obtained scores were 0.9324 for S1464I, 0.9870 for A1705V, and 0.9490 for T1602I (see Table 1).

### 3.6 Protein–protein docking measures of NOTCH1 and mutants

**FIGURE 5:**
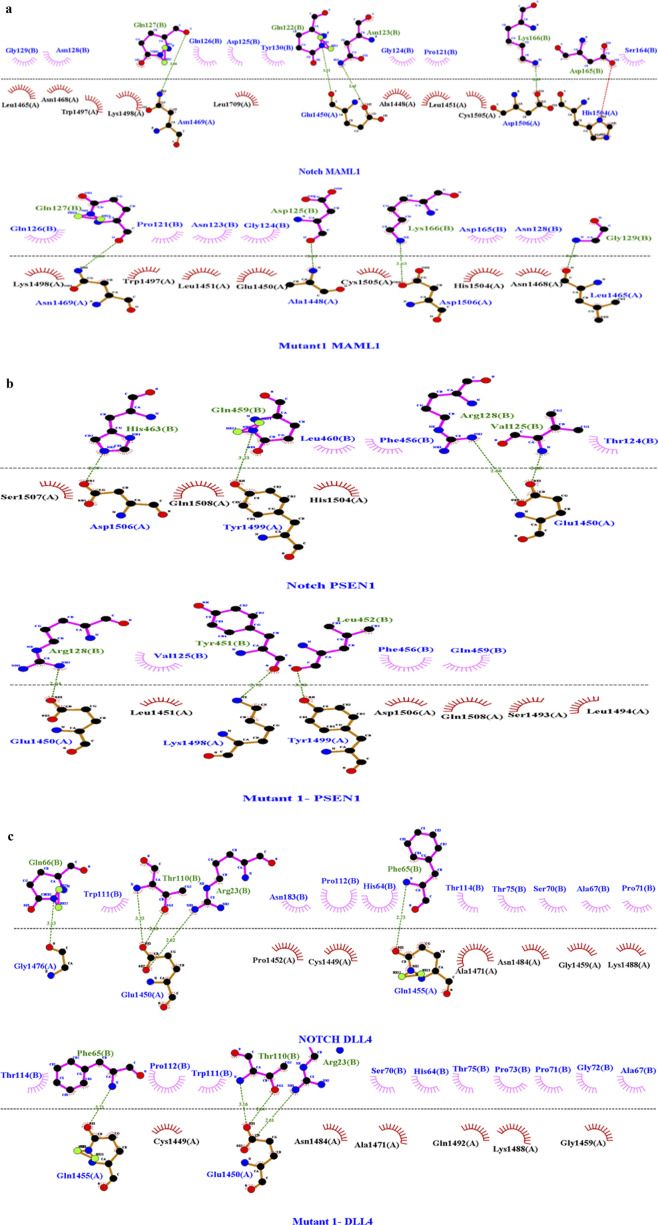
**(A)** Molecular docking interaction analysis of wild-type with MAML1 and mutant 1 with MAML1 with a Z-score of −1.9. Among the bonds, the green dashed line in the image depicts the hydrogen bond, red dashed lines indicate the salt bridges, purple indicates the external bonds, orange depicts the interface bond 1, and stellations represent the van der Waals interaction. Here, in the pictorial representation, the WT interaction has four hydrogen bonds and one salt bridge, while the mutant interaction resulted in the removal of that salt bridge with four hydrogen bonds. The dots in different colors indicate the atoms: blue indicates nitrogen atoms, black indicates carbon atoms, and red indicates oxygen atoms. **(B)** Molecular docking interaction analysis of wild-type with PSEN1 and mutant 1 with PSEN11 with a Z-score of −2.0. Among the bonds, the green dashed line in the image depicts the hydrogen bond, and stellations represent the van der Waals interaction. Here, in the pictorial representation, the WT interaction has four hydrogen bonds with no salt bridges, and the mutant interaction has reduced the hydrogen bonds to three with no salt bridges. The dots in different colors indicate the atoms: blue indicates nitrogen atoms, black indicates carbon atoms, and red indicates oxygen atoms. **(C)** Molecular docking interaction analysis of wild-type with DLL4 and mutant 1 with DLL 4 with a Z-score of −1.7. Among the bonds, the green dashed line in the image depicts the hydrogen bond, and stellations represent the van der Waals interaction. Here, in the pictorial representation, the WT interaction has five hydrogen bonds with no salt bridges, while the mutant interaction has reduced the hydrogen bonds to three with no salt bridges. The dots in different colors indicate the atoms: blue indicates nitrogen atoms, black indicates carbon atoms, and red indicates oxygen atoms.

The docking of NOTCH1 and its mutants was carried out in the HADDOCK 2.4 server. Docking analysis indicated that all six interactors of NOTCH1 successfully formed the complexes. Among the obtained scores, the HADDOCK and Z-scores were taken into consideration to identify the best model for further dynamics simulation. Among the three mutants, mutant 1 (S1464I) with the interactors MAML1, PSEN1, and DLL4 shows more binding affinity in terms of the Z-score (−1.9, −2.0, and −1.7) and HADDOCK score (−59.7–55.4, and −64.3). The interaction analysis with mutant 2 resulted in a higher binding affinity with interactors such as MAML1, JAG2, and DLL4, with Z-scores of −1.7, −1.7, and −1.9 and HADDOCK scores of −64.6, –79.9, and −61.8. The mutant 3 docking score shows better interaction with the interactors MAML1 and PSEN1, with a Z-score of −1.4 and JAG2, JAG1, and DLL4 with a Z-score of −1.5. The HADDOCK scores of MAML1 and PSEN1 with mutant 3 are −62.7 and −55.4, respectively. The obtained HADDOCK scores for the interactors JAG2, JAG1, and DLL4 for mutant 3 are −79.0, −68.7, and −63.1, respectively. The interaction analysis showed that mutant 1 can be selected for further studies in terms of binding energy. With the interaction of MAML1, the wild-type interactor has four hydrogen bonds of lengths 3.06, 3.28, 2.68, and 2.72 with one salt bridge. The mutant 1 interaction with MAML1 resulted in the same number of hydrogen bonds with the removal of the existing salt bridge. The interactor PSEN1 resulted in four hydrogen bonds during the interaction with the wild-type, with lengths 2.69, 3.21, 2.66, and 2.60, but the mutant 1 interaction with PSEN1 resulted in a reduction in the existing number of hydrogen bonds to 3. The DLL4 interaction with the wild-type resulted in five hydrogen bonds, with lengths 3.13, 3.33, 2.63, 2.62, and 2.73, while the DLL4 interaction with the mutant resulted in a reduction in the number of hydrogen bonds to 4. The detailed outputs of the docking measures are summarized in Table 3, Figures 5a–c, and Supplementary Table S3.

**TABLE 3:** Estimated molecular docking measures (Z-scores) HADDOCK for wild-type, mutant 1 (S1464I), mutant 2 (T1602I), and mutant 3 (A1705V). Mutant 1 shows higher binding affinity with interactors such as MAML1, PSEN1, and DLL4 with the Z-scores of −1.9, −2.0, and −1.7, respectively. Average of Z-scores.

Z- scores	MAML1	PSEN1	MAML2	JAG2	JAG1	DLL4	Average score
NOTCH1	−1.8	−1.8	−1.3	−1.3	−1.7	−1.5	−1.5
Mutant 1	−1.9	−2.0	−1.4	−1.5	−1.4	−1.7	−1.6
Mutant 2	−1.7	−1.4	−1.2	−1.7	−1.6	−1.9	−1.5
Mutant 3	−1.4	−1.4	−1.3	−1.5	−1.5	−1.5	−1.4

### 3.7 Molecular dynamics simulation analysis

**FIGURE 6:**
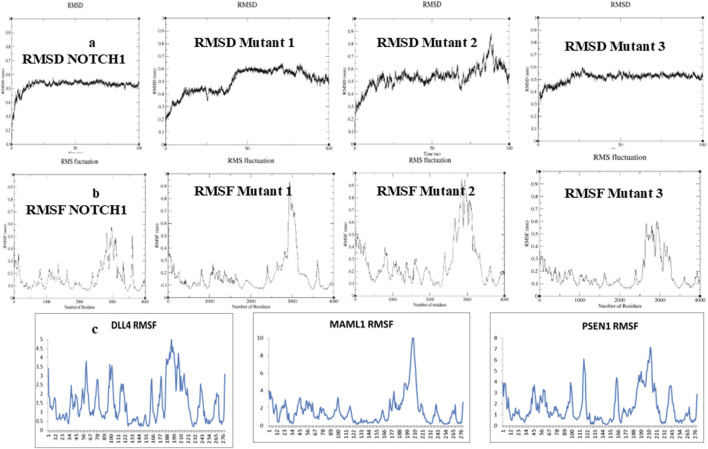
**(a–c)** Molecular dynamics simulation of native protein NOTCH1, mutant 1 (S14641), mutant 2 (T16021), and mutant 3 (A1705V). The average RMSD and RMSF for mutant 1 (S14641) were found to be more than those of mutant 2 (T16021), mutant 3 (A1705V), and the native protein NOTCH1. The obtained RMSD (6a) and RMSF (6b) values of mutant 1 are 0.49 and 0.17, respectively. The RMSF (6c) values of the docked complexes of mutant 1 with the interactors are 1.45 (DLL4), 1.64 (MAML1), and 1.63 (PSEN1).

**FIGURE 7:**
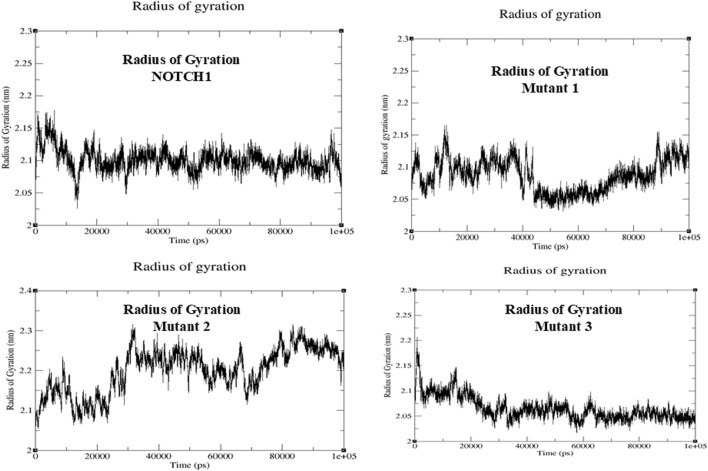
Molecular dynamics simulation of native protein NOTCH1, mutant 1 (S14641), mutant 2 (T16021), and mutant 3 (A1705V). The radius of gyration analysis was carried out to identify the compactness of the protein. The Rg analysis results showed a higher value of 2.199 for mutant 1, 2.08 for mutant 2, 2.06 for mutant 3, and 2.1 for native NOTCH1, which suggests a more stable and compact protein–protein complex formation compared to that of other mutants.

MD simulation analysis was carried out for NOTCH1 and its interactors MAML1, PSEN1, and DLL4 in terms of RMSD, RMSF, and the radius of gyration (Rg). During simulation, the RMSD determines the conformation of the protein by evaluating structural stability and estimating the average distance between the backbone atoms of a protein. Among the obtained RMSD values, the average RMSD value for mutant 1 (S1464I), i.e., 0.49, was found to be more than that of mutant 2 (T1602I), mutant 3 (A1705V), and the native protein NOTCH1. The RMSD plots and scores show high structural deviation compared to the native NOTCH1 and the other mutants, which suggests greater conformational flexibility upon the mutation. This results in the mutant 1 S1464I substitution potentially inducing notable changes in the NOTCH1 backbone that may alter its structural integrity. While computing the RMSF, both the native protein and the residual mobility of each protein–protein combination are taken into consideration for the assessment. The RMSF plots show localized peaks corresponding to specific regions with enhanced atomic fluctuations. These regions represent flexible loops or domains that are involved in inter-domain communication. The elevated fluctuations imply altered dynamics that could influence the signaling pathway and the activation of the NOTCH1 downstream signaling. The average RMSF values were higher in mutant 1 compared to the native, mutant 2, and mutant 3 proteins. The resulting RMSF value of mutant 1 (S1464I) is 0.17, and among the interactors, MAML1 and PSEN1 show the high average RMSF values of 1.64 and 1.63. The higher RMSF values indicate increased flexibility in the binding interfaces, suggesting that mutationally induced alterations in NOTCH1 may propagate to its interactors, potentially weakening or altering the binding affinity and downstream signaling efficiency. The observed alterations in the mutants, particularly mutant 1(S1464I), imply that these substitutions may disrupt structural dynamics and modulate the functional behavior of the NOTCH signaling complex (Figures 6a–c). The level of compactness and overall structural stability in the native protein and its interactor’s structure were determined using RG analysis. The Rg analysis resulted in the mutant 1 having a higher value of 2.199 and more fluctuations, which may lead to alterations in the structural and functional integrity of NOTCH1. The observed differences indicate that the S1464I mutation most strongly perturbs the structural cohesion of NOTCH1, potentially affecting its protein–protein interaction efficiency and signaling fidelity. Overall, regions showing higher Rg fluctuations likely represent flexible regions that could compromise the structural stability and biological activity of NOTCH1 (Figure 7).

## 4 Discussion

Lung adenocarcinoma, as a histological subtype of NSCLC, remains the most prominent and malignant tumor with a poor survival rate and a substantial mortality rate. Approximately 40% of lung cancer cases account for LUAD, with an average 5-year survival rate of 15% (Wang et al., 2023). The considerable genetic and molecular heterogeneity of LUAD signifies a cause for concern in the prognostic and treatment realms. Over the past few years, research on treating LUAD has focused heavily on predicting genes associated with prognosis, important genes in immunotherapies, and important signaling pathways in progression (Wang et al., 2023). Nonetheless, the high rate of susceptibility to drug resistance tends to remain the main challenge and the key reason for the high mortality rate in LUAD. Therefore, there is a need to find novel molecular markers and prognostic models to stratify and tailor treatment regimens for patients with lung adenocarcinoma. Triggering of alteration in cellular metabolism, along with divergent activation of key signaling pathways such as EMT, Notch signaling, and their interplay, plays a key role in the initiation and advancement of carcinogenesis. Together with the changes and alterations are critical shifts in tumor development control and the development of resistance against chemotherapy. As a whole, these characteristics are widely acknowledged as the central hallmarks of cancer.

It is speculated that numerous types of stimuli, signal transduction pathways, and transcription factors influence and regulate the mechanisms that control the acquisition of EMT. The Notch signaling pathway has recently been shown to be a vital regulator in the production of EMT (Niessen et al., 2008; Sahlgren et al., 2008; Timmerman et al., 2004; Zavadil et al., 2004) and a key player in cross-talk during LUAD progression (John and Sudandiradoss, 2024a). When Notch signaling is activated in endothelial cells, it effectively impacts morphological, physical, and structural changes, which may consequently lead to mesenchymal activation. These changes include the downregulation of endothelial markers (VE-cadherin, Tie1, Tie2, platelet–endothelial cell adhesion molecule-1, and endothelial NO synthase), upregulation of mesenchymal markers (α-SMA, fibronectin, and platelet-derived growth factor receptors), and migration toward PDGF-B driven processes (Noseda et al., 2004). Thus, it is speculated that only cells expressing activated Notch can undergo Notch-induced EMT. Furthermore, it is known that Jagged-1 stimulation of endothelial cells causes a comparable mesenchymal transformation, indicating that Jagged-1-mediated Notch signaling activation is crucial for the development of EMT (Wang et al., 2010).

In the realm of precision medicine, *in silico* predictions are becoming increasingly popular as an approach to detecting and assessing nsSNP-associated IDRs and post translational modifications that could affect drug metabolism, metabolic pathways, and protein–protein interactions (Sunil Krishnan et al., 2021). Therefore, the ruled-out SNPs can potentially serve a prognostic role in the diagnosis field. Despite changes in functional characteristics, molecular alterations, and metabolic pathways, the identification and characterization of harmful nsSNPs, IDRs, and IDRs with post-translational modifications can lead to the hallmarks of cancer (Deng et al., 2024; Guo et al., 2023; Liu et al., 2023). Therefore, the goal of this current research was to find the deleterious SNPs in NOTCH1 and figure out how these SNPs had an impact on LUAD progression. Using *in silico* prediction techniques, the structural and functional alterations in NOTCH1 genes have been documented. The knowledge gleaned from these analyses might be very helpful for further NOTCH1 research studies. Integrating many techniques offers a useful insight into the identification and characterization of nsSNPs as a single-tool prediction often cannot yield a correct result (Sukumar et al., 2021). We, therefore, combined sequence- and structure-based *in silico* techniques to evaluate and assess the detrimental effects of the nsSNPs in NOTCH1. Thus, the collected nsSNPs from the NCBI and COSMIC databases have been evaluated using prediction tools, resulting in three stable SNPs with a ΔΔ*G* value of 0.62 for S1464I, 0.13 for A1705V, and 0.56 kcal/mol for T1602I.

The identified deleterious mutants S1464I, A1705V, and T1602I were located in the juxtamembrane and negative regulatory region (NRR) and the N-terminus region of the NICD domain in the NOTCH1 protein. The mutants S1464I and T1602I are located near or within the juxtamembrane or the NRR region, while the mutant A1705V is located at the N-terminus region of the NICD, which is close to the RAM domain that mediates the transcriptional complex assembly. Although the positional context of these substitutions suggests that they may collectively influence both receptor activation and intracellular signaling in the pathway, the mutant S1464I is positioned within the juxtamembrane or the NRR region. Here, in this mutation, the serine is replaced with isoleucine, which alters the hydrogen bond networks and phosphorylation sites, reducing the receptor activation in the signaling pathway and thereby enhancing downstream signaling. The T1602I mutant is located adjacent to the NICD boundary and may similarly affect phosphorylation-dependent regulation and S3-mediated cleavage, which alter the structural dynamics of protein stability and efficiency and generation of the NICD domain. In contrast, the mutant A1705V is located at the onset of the NICD region near the RAM domain, which directly impacts the signaling core of NOTCH1. This region mediates the assembly of the NICD–CSL–MAML complex, which is a hotspot for the regulatory turnover.

Together, these three stabilizing mutants may act cooperatively to enhance NOTCH1 signaling by decreasing receptor degradation, promoting NICD accumulation, and strengthening transcriptional activation. This cumulative stabilization could convert transient ligand-dependent Notch signaling into the ligand-independent type, thus driving malignant transformation and progression. In lung adenocarcinoma, sustained NICD signaling has been shown to promote the EMT pathway, cancer stemness, metabolic reprogramming, and therapeutic resistance, with a combined effect of the KRAS and EGFR pathways. Thus, the spatial positioning and biophysical impact of these mutations provide a mechanistic framework linking enhanced NOTCH1 structural stability to deregulated signaling output.

Other than the deleterious SNPs, genetic alterations and post-translational modifications are also directly linked to tumorigenesis. Thus, to identify the role of IDRs and the PTMs in the human *NOTCH1* gene, the sequence was subjected to *in silico* prediction tools such as IUPred 3 and MusiteDeep. Using an integrated computational approach, we identified 10 types of post-translational modifications, such as hydroxyproline, O-linked glycosylation, phosphothreonine, phosphoserine, N-linked glycosylation, phosphotyrosine, pyrrolidine–carboxylic acid, SUMOylation, ubiquitination, N6-acetyllysine, methyllysisne, and methylariginine in the NOTCH1 sequence. The IUPred 3 and ANCHOR2 prediction results identify the IDR region from sequence position 1,600 in parts, indicating that these regions can undergo a disorder-to-order transition upon binding to another protein, which affects functional and structural changes. Moreover, the IDR regions mainly fall in the category of ankyrin repeats of the NOTCH1 protein. IUPred 3 also predicts the Pfam information’s sequence with the functional domains, and in our analysis, the NOTCH1 sequence resulted in the functional domains such as the EGF domain of residues 63–134, the Notch domain of residues 1,446–1,562, EGF_CA, HEGF, the NOD family domain of residues 1,566–1,729, the ANK_2 repeat 1,990–2,092, DUF3454 of residues 2,479–2,540, the PEST domain of residues 2,478–2,541, and phosphorylation-dependent degradation sites 2,508–2,515.

Many studies have reported that pathogenic SNPs in the functional and conserved regions of a protein impact the expression of that gene, its structure, its stability, and functional changes that may lead to disorders such as cancers (Shastry, 2009). Thus, from our results, we can infer that the identified deleterious SNPs are in the conserved domain, having a score of 8–9, inferring that they are strong candidates that can alter the normal functioning of NOTCH1 and can lead to LUAD progression. The identified SNP S1464I falls in the NOTCH domain (NICD domain), which is a functional domain of the NOTCH protein that helps in the transcription of target genes and the EMT pathway. The other two SNPs T1602I and A1705V fall in the NOD domain and NODP domain, respectively, having a main function in Notch signaling and cell fate determination. HOPE analysis revealed that the stabilizing SNPs S1464I, T1602I, and A1705V occur within conserved regions of the NOTCH1 and NOD domains, suggesting structural and functional importance (Kopan and Ilagan, 2009). The S1464I mutation replaces a polar serine with a hydrophobic isoleucine, potentially disrupting phosphorylation or hydrogen bonding and stabilizing the juxtamembrane/NRR region, which may enhance receptor persistence or ligand sensitivity (Louvi and Artavanis-Tsakonas, 2006). T1602I, near the γ-secretase cleavage site, introduces a bulkier isoleucine that could rigidify the local environment, improving NICD release and promoting sustained signaling (Gordon et al., 2008) A1705V, located within the NICD RAM domain, may strengthen hydrophobic packing, enhance CSL/MAML interactions, and reduce NICD turnover (Oswald and Kovall, 2018).

Collectively, the physicochemical alterations introduced by these three stabilizing SNPs—increased hydrophobicity, loss of polar/charged residues, and local side-chain enlargement—are predicted to reinforce domain integrity, reduce conformational plasticity, and enhance NICD persistence. Functionally, these effects may synergize to prolong NOTCH1 signaling output even under low-ligand conditions, thereby promoting key oncogenic traits in LUAD, such as stemness, survival under stress, and resistance to targeted therapies (Westhoff et al., 2009; Zhu et al., 2019; Bray, 2016). Thus, the integration of HOPE-derived biophysical insights with domain-level functional mapping suggests that these stabilizing variants may shift NOTCH1 signaling dynamics toward a constitutively active, pro-tumorigenic state.

The molecular docking and dynamics results revealed that the stabilizing mutations, particularly mutant 1(S1464I), markedly influence the structural stability and interaction dynamics of NOTCH1 with its key partners MAML1, PSEN1, and DLL4. The RMSD and Rg plots showed that S1464I exhibits higher average deviations and slightly increased compactness compared to native NOTCH1, indicating a conformationally stabilized yet dynamically restrained structure. The RMSF plots demonstrated reduced flexibility at several functional regions of mutant 1, suggesting altered local motions that could modulate protein–protein interface stability. Consistent with docking data, these conformational changes likely enhance the binding affinity of mutant 1 toward DLL4, MAML1, and PSEN1, stabilizing the Notch receptor–ligand transcriptional complexes. Such stabilization may prolong NICD availability and transcriptional signaling, thereby abnormally sustaining Notch activation. Persistent Notch signaling can crosstalk with EMT regulatory networks, promoting cellular plasticity, invasion, and tumor progression in LUAD. Thus, the combined structural compactness, altered hydrogen-bond dynamics, and enhanced interactor binding predicted for the mutants, especially S1464I, may underpin aberrant pathway activation and oncogenic transformation.

The mapping of the degron analysis resulted in 40 degrons, in which two experimentally validated phosphodegrons were present in the PEST domain of NOTCH1 with an IDR disordered region. The PEST domain region of NOTCH1 is mainly engaged in the regulation of protein stability, and the presence of phosphodegrons in this domain (sequences that promote degradation via phosphorylation) might contribute to the destabilization of NOTCH1. In the context of LUAD, these mutations or alterations in phosphodegrons may either impair the degradation process or make the protein more susceptible to degradation and can lead to dysregulation of Notch signaling, leading to abnormal accumulation or loss of NOTCH1 activity (Westhoff et al., 2009). This interference in NOTCH1 signaling can disrupt normal cellular functions, such as differentiation and stem cell-like properties, and promote tumorigenesis and progression in lung adenocarcinoma (Zong et al., 2016; Liu et al., 2021).

The mutations in the disordered region and the associated post-translational modifications can also be a factor for tumor progression (Keith Dunker et al., 2002; Li W. et al., 2024). The two main PTMs identified in the NOTCH1 protein were O-linked glycosylation and phosphothreonine, which correlate with IDR regions in NOTCH1. Post-translational modifications, such as O-linked glycosylation and phosphothreonine, play a key role in modulating proteins functional stability and interactions with other proteins. O-linked glycosylation is considered more complicated than N-linked as the consensus sequence for its initiation is unknown, and many O-glycosylation-decorated proteins play an important role in cancer-associated biological processes (Kaur et al., 2013). The reported studies state that the key player or core player of O-glycan’s C1GALT1 plays an important role in tumor progression in lungs through the phenotypic change induction of the EMT signaling pathway (Gupta et al., 2020). Yamamoto (2020) reported through his study that O-glycosylation of Notch has numerous unique and overlapping roles in Notch signaling. The PTM O-linked glycosylation can impact proteins’ conformation, stability, and trafficking, potentially resulting in dysregulated NOTCH1 signaling that promotes tumor growth. Altered glycosylation patterns are frequently observed in cancer cells and can contribute to the disrupted activity of key regulators of cell signaling. The *in silico* analysis of NOTCH1 revealed O-linked glycosylation and intrinsically disordered regions at the same sites, and the crosstalk with the EMT signaling pathway may the reason for the oncogenic role of NOTCH1 in LUAD tumorigenesis. Phosphothreonine phosphorylation occurring on threonine was the next major PTM identified in the NOTCH1 sequence. PTMs are an integral part of the tumor cell’s adaptation and response to intracellular and environmental changes. In the case of NOTCH1, these modifications might interrupt its ability to interact with other signaling molecules or transcription factors that are crucial for regulating cell-fate decisions. Phosphothreonine modifications in the NOTCH1 protein may influence the activation or inhibition of the NOTCH1 signaling cascade, leading to uncontrolled proliferation or resistance to apoptosis. From the conveyed findings, we can anticipate that the serine/threonine kinases at the phosphorylation site are the probable responsible kinases for the crosstalk between signaling pathways and other alterations (Geffen et al., 2023). Considering this, we can speculate from our *in silico* research that the oncogenic involvement of NOTCH1 in the progression of LUAD may be due to the presence of phosphothreonine, the PTM alteration that permits crosstalk signaling between NOTCH1 and EMT.

In lung adenocarcinoma, fluctuations in NOTCH1 activity are mainly caused by mutations, post-translational modifications, and truncations that may drive the transition from an epithelial to a mesenchymal phenotype, which leads to the increased metastatic ability of the cancer cell. This change plays a key role in promoting tumor cell migration, invasion, and drug resistance. The critical drivers of the EMT signaling pathway are Snail, Slug, and Twist, which may have been influenced by NOTCH signaling, and dysregulated NOTCH1 signaling in lung adenocarcinoma can, therefore, drive EMT and subsequent metastasis.

The collective changes of these molecular disruptions in NOTCH1, mainly the presence of destabilizing SNPs, phosphodegrons, post-translational modifications, IDR regions, and crosstalk with EMT signaling, create an impact on the dysregulation of Notch signaling. The structural and functional integrity of NOTCH1 is mainly controlled by a complex interplay between its coding sequence, intrinsically disordered regions, and post-translational modifications that coordinate its activation, turnover, and transcriptional activity. Deleterious SNPs within the critical NOTCH1 domain can alter this regulatory equilibrium, causing changes in amino acid chemistry, local folding of the protein, and the landscape of PTM motifs. The presence of the IDRs within the Notch intracellular domains makes the protein more flexible. This flexibility of the protein is important as it allows the protein to interact with other molecules and undergo modifications, such as tagging with a phosphate group or ubiquitin, and passing the signal along to the inside of the cell. These regions are very flexible and sensitive to changes in the amino acid sequence, undergoing an order-to-disorder transition, which alters the protein, disrupts signaling and can lead to diseases such as cancer. PTMs act as a molecular switch that fine-tunes NOTCH1 activation and stability. Phosphorylation within the regulatory motifs not only facilitates proteolytic activation but also serves as a trigger for ubiquitination through phosphodegrons. The loss or alteration of these phosphodegrons through deleterious SNPs and IDRs disrupts E3 recognition, leading to impaired ubiquitination.

The cumulative outcome of these molecular perturbations is a shift from transient, ligand-dependent signaling toward persistent, ligand-independent activation of the NOTCH1 pathway. In the context of lung adenocarcinoma, this stabilization-driven hyperactivation enhances the oncogenic phenotypes, including the EMT pathway, cancer stem-cell maintenance, metabolic reprogramming, and therapeutic resistance. Altered NOTCH signaling further promotes crosstalk with the KRAS and EGFR signaling pathways, amplifying tumor proliferation and invasion while remodeling the tumor microenvironment to favor immune evasion and angiogenesis. Collectively, the deleterious SNPs, altered IDRs, PTM sites, and phosphodegrons’ synergistic effect transform NOTCH1 from a tightly regulated developmental signal transducer into a chronically active oncogenic driver in LUAD.

Taken together, disruptions in NOTCH1 signaling play a key role in the progression of lung adenocarcinoma by modulating normal cellular communication, promoting tumor development, invasiveness, and metastasis, and possibly contributing to drug resistance. Understanding these mechanisms and gaining insights into these pathways can help identify new therapeutic strategies, including Notch inhibitors and treatments targeting EMT, to better control and manage the progression of lung adenocarcinoma.

## 5 Conclusion

The present investigation indicates that Notch signaling has evidently emerged as a critical pathway in lung cancer, especially in LUAD. It is plainly appreciated that aberrant Notch signaling, especially NOTCH1, contributes to the pathophysiology of LUAD. A deeper understanding in this study shows that the role of NOTCH1 in LUAD progression is very complex. Even though NOTCH1 is downregulated in LUAD, it can still serve as a prognostic marker and TSG for LUAD patients. Moreover, NOTCH1 acts as a key player in Notch signaling and the crosstalk of the EMT signaling pathway, which helps in LUAD proliferation and metastasis. Thus, high NOTCH1 activation in NSCLC might lead to a worse prognosis and treatment resistance. The presence of deleterious SNPs within the functional domain of NOTCH1 plays a potential role in disrupting the normal function of NOTCH1 and contributes to LUAD progression. Moreover, for genetic alterations, PTMs, such as O-linked glycosylation and phosphothreonine modifications, along with the phosphodegrons in the PEST domain, play a key role in governing NOTCH1 function and its interactions with various cellular pathways, including the EMT, which is a pivotal process in cancer metastasis. Therefore, our study highlights the complex and multifaceted role of NOTCH1 in LUAD, underscoring its potential as a therapeutic target within Notch signaling. Importantly, the identified deleterious SNPs, PTMs, particularly within the PEST domain, and other degradation determinants, may critically influence NOTCH1 function and stability, and it is essential to experimentally verify the functional impact of these predicted SNPs and PTM sites to better understand their biological significance. Furthermore, exploring therapeutic strategies aimed at these specific allosteric sites and regulatory mechanisms of NOTCH1 degradation could open new avenues for targeted treatment, and validating the prognostic value of these biomarkers in larger clinical cohorts will be crucial to confirming their utility in patient stratification and personalized medicine. These future directions offer promising opportunities to translate our findings into clinical benefit for LUAD patients.

## Data Availability

The datasets presented in this study can be found in online repositories. The names of the repository/repositories and accession number(s) can be found in the article/Supplementary Material.
