# Supplementary material for: Neurogenic locus notch homolog protein 1 (NOTCH 1) SNP informatics coupled with intrinsically disordered regions and post-translational modifications reveals the complex structural crosstalk of Lung Adenocarcinoma (LUAD)
Source: Front Bioinform. 2025 Dec 10;5:1641521. doi: 10.3389/fbinf.2025.1641521 (PMC12727990; doi:10.3389/fbinf.2025.1641521)
Supplement: Supplementary file 1 [file Table1.docx]

**Supplementary Table 1: The table depicts the Sequence based prediction of Deleterious SNPs in NOTCH1.**

| **SNP** | **Amino acid position and substitution** | **SIFT prediction with score** | **POLYPHEN2**  **prediction with score** | **Predict-SNP prediction with score** | **SNP&GO prediction with score** | **PANTHER prediction with score** |
| --- | --- | --- | --- | --- | --- | --- |
| rs199652954 | R1926C | Deleterious- 0.001 | Probably Damaging- 0.995 | Disease | Neutral  RI=5 | Probably damaging =0.74 |
| rs199860726 | A1570G | Deleterious-0.028 | Benign-0.261 | Neutral | Neutral  RI=9 | Probably damaging =0.74 |
| rs200245794 | P1257L | Deleterious-0.001 | Possibly Damaging- 0.802 | Disease | Neutral  RI=9 | Probably damaging = 0.78 |
| rs200520088 | T349P | Deleterious-0.003 | Probably Damaging- 1.000 | Disease | Disease RI=9 | Probably damaging = 0.85 |
| rs200562991 | T432M | Deleterious-0.001 | Probably Damaging- 1.000 | Disease | Neutral  RI=7 | Probably damaging = 0.74 |
| rs200893930 | R2549C | Deleterious-0.02 | Benign-0.326 | Neutral | Neutral  RI=9 | Possibly damaging = 0.5 |
| rs201058656 | N2086S | Deleterious-0.019 | Benign-0.164 | Neutral | Neutral  RI=7 | Probably damaging = 0.74 |
| rs201163739 | S1004L | Deleterious-0.003 | Benign-0.327 | Neutral | Neutral  RI=9 | Possibly damaging = 0.5 |
| - | S1464I | Deleterious-0.00 | Possibly Damaging- 0.494 | Disease | Disease RI=9 | Possibly damaging = 0.5 |
| rs201236538 | A348P | Deleterious-0.003 | Possibly Damaging- 0.880 | Disease | Disease RI=1 | Probably damaging = 0.57 |
| rs201620358 | R912W | Deleterious-0.017 | Possibly Damaging- 0.849 | Disease | Neutral  RI=8 | Possibly damaging = 0.5 |
| rs201779159 | L1805P | Deleterious-0.019 | Probably Damaging- 0.935 | Disease | Neutral  RI=9 | Possibly damaging = 0.5 |
| rs202145498 | T311P | Deleterious-0.011 | Possibly Damaging- 0.597 | Disease | Neutral  RI=5 | Probably damaging = 0.85 |
| rs367586502 | N918S | Deleterious-0.013 | Benign-0.260 | Neutral | Neutral  RI=9 | Probably damaging = 0.85 |
| rs367860186 | T1006I | Deleterious-0.017 | Probably Damaging- 0.962 | Disease | Neutral  RI=9 | Possibly damaging = 0.5 |
| rs368396893 | V1750M | Deleterious-0.02 | Benign-0.410 | Neutral | Neutral  RI=9 | Possibly damaging = 0.5 |
| rs369721921 | V276M | Deleterious-0.002 | Probably damaging = 0.984 | Disease | Neutral  RI=6 | Probably damaging = 0.74 |
| rs369730402 | V1096M | Deleterious-0.002 | Probably damaging = 0.941 | Disease | Neutral  RI=9 | Possibly damaging = 0.5 |
| - | A1705V | Deleterious-0.01 | Probably damaging = 0.998 | Disease | Disease RI=9 | Probably damaging = 0.74 |
| rs369915496 | T1491M | Deleterious-0.01 | Possibly Damaging- 0.503 | Disease | Neutral  RI=9 | Possibly damaging = 0.5 |
| rs370797169 | R955C | Deleterious-0.012 | Possibly Damaging- 0.893 | Disease | Neutral  RI=8 | Possibly damaging = 0.5 |
| rs371050668 | P877L | Deleterious-0.001 | Probably damaging = 1.000 | Disease | Disease RI=9 | Probably damaging = 0.85 |
| rs371333249 | G275S | Deleterious-0.009 | Probably damaging = 1.000 | Disease | Disease RI=2 | Probably damaging = 0.74 |
| rs371414501 | A1944T | Deleterious-0.002 | Possibly Damaging- 0.792 | Disease | Neutral  RI=7 | Probably damaging = 0.78 |
| rs371742334 | R2159C | Deleterious-0.001 | Probably damaging = 0.939 | Disease | Neutral  RI=9 | Probably damaging = 0.74 |
| rs372698234 | P2137S | Deleterious-0.043 | Possibly Damaging- 0.848 | Disease | Neutral  RI=9 | Probably damaging = 0.57 |
| rs373806373 | R2087W | Deleterious-0.001 | Probably damaging = 0.994 | Disease | Disease RI=4 | Probably damaging = 0.85 |
| rs373841359 | R1758H | Deleterious-0.032 | Probably damaging = 0.939 | Disease | Neutral  RI=9 | Possibly damaging = 0.5 |
| rs374434131 | G766V | Deleterious-0.001 | Probably damaging = 1.000 | Disease | Disease RI=9 | Probably damaging = 0.85 |
| - | T1602I | Deleterious-0.04 | Probably damaging = 0.999 | Disease | Disease RI=9 | Probably damaging = 0.74 |
| rs374453977 | R2313W | Deleterious-0.006 | Probably damaging = 0.999 | Disease | Disease RI=8 | Probably damaging = 0.74 |
| rs374787627 | A1935V | Deleterious-0.002 | Possibly Damaging- 0.600 | Disease | Neutral  RI=5 | Probably damaging = 0.78 |
| rs375018022 | R1633H | Deleterious-0.019 | Possibly Damaging- 0.863 | Disease | Neutral  RI=9 | Possibly damaging = 0.5 |
| rs375190395 | S1168F | Deleterious-0.042 | Probably damaging = 0.962 | Disease | Disease RI=7 | Possibly damaging = 0.5 |
| rs375969725 | R2120C | Deleterious-0.002 | Probably damaging = 0.996 | Disease | Disease RI=7 | Probably damaging = 0.57 |
| rs376055493 | T559M | Deleterious-0.011 | Probably damaging = 0.927 | Disease | Disease RI=7 | Possibly damaging = 0.5 |
| rs376104770 | P284L | Deleterious-0.001 | Probably damaging = 0.912 | Disease | Neutral  RI=5 | Probably damaging = 0.74 |
| rs376799353 | A1471V | Deleterious-0.018 | Possibly Damaging- 0.755 | Disease | Neutral  RI=9 | Possibly damaging = 0.5 |
| rs376902925 | T211A | Deleterious-0.034 | Benign-0.423 | Neutral | Neutral  RI=9 | Possibly damaging = 0.5 |
| rs377289044 | C1284W | Deleterious-0.000 | Probably damaging = 0.999 | Disease | Neutral  RI=7 | Probably damaging = 0.85 |
| rs377294245 | V1739M | Deleterious-0.004 | Benign-0.230 | Neutral | Neutral  RI=9 | Possibly damaging = 0.5 |
| rs377343669 | T961M | Deleterious-0.02 | Probably damaging = 1.000 | Disease | Neutral  RI=9 | Probably damaging = 0.74 |
