# Supplementary figures and images for: Neurogenic locus notch homolog protein 1 (NOTCH 1) SNP informatics coupled with intrinsically disordered regions and post-translational modifications reveals the complex structural crosstalk of Lung Adenocarcinoma (LUAD)
Source: Front Bioinform. 2025 Dec 10;5:1641521. doi: 10.3389/fbinf.2025.1641521 (PMC12727990; doi:10.3389/fbinf.2025.1641521)

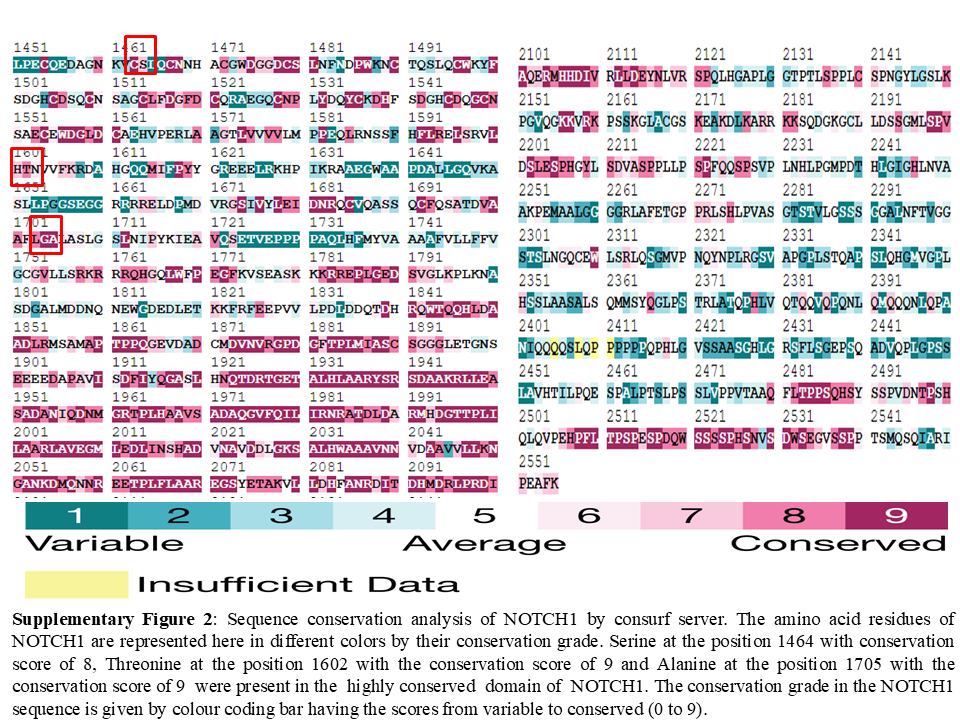

Supplement: Supplementary file 2 [file Image2.tif]

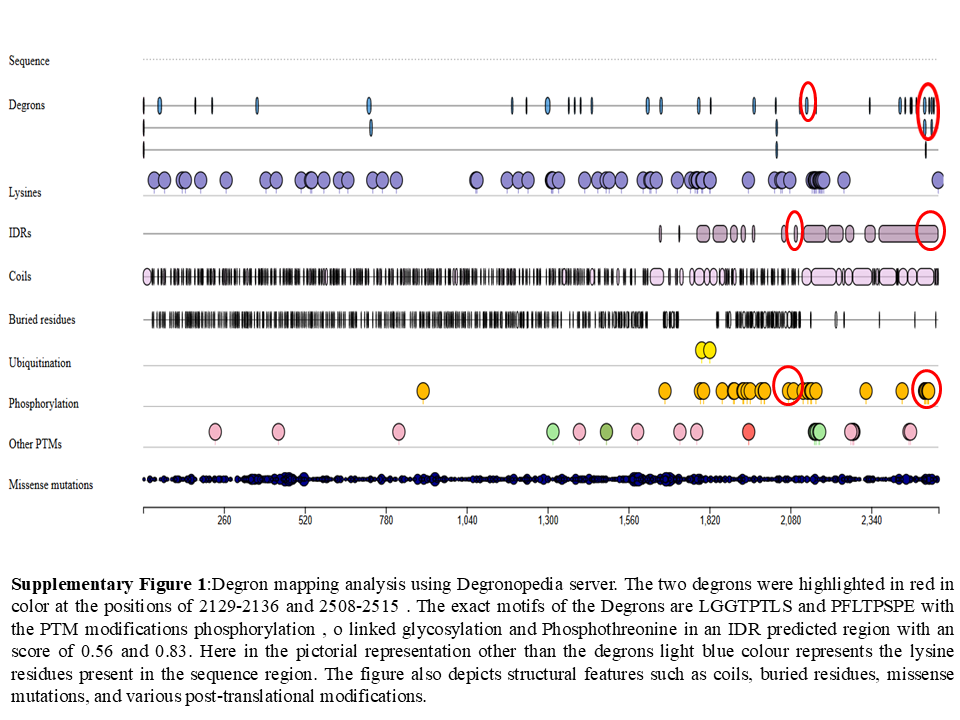

Supplement: Supplementary file 3 [file Image1.tif]
