# Supplementary material for: Neurogenic locus notch homolog protein 1 (NOTCH 1) SNP informatics coupled with intrinsically disordered regions and post-translational modifications reveals the complex structural crosstalk of Lung Adenocarcinoma (LUAD)
Source: Front Bioinform. 2025 Dec 10;5:1641521. doi: 10.3389/fbinf.2025.1641521 (PMC12727990; doi:10.3389/fbinf.2025.1641521)
Supplement: Supplementary file 4 [file Table2.docx]

**Supplementary Table 2: Post- Translational Modification (PTM) Predictions in NOTCH1 using MusiteDeep. The regions having the PTM modification with an average score of above0.5 are provided in the table with their position number and amino acid residue.**

| **ID** | **Position** | **Residue** | **PTM scores** |
| --- | --- | --- | --- |
| sp\|P46531\| | 55 | P | Hydroxyproline:0.587 Hydroxyproline:0.587 |
| sp\|P46531\| | 60 | P | Hydroxyproline:0.647 |
| sp\|P46531\| | 65 | S | Phosphoserine:0.169;O-linked_glycosylation:0.887 |
| sp\|P46531\| | 66 | T | Phosphothreonine:0.632;O-linked_glycosylation:0.278 |
| sp\|P46531\| | 73 | T | Phosphothreonine:0.084;O-linked_glycosylation:0.886 |
| sp\|P46531\| | 108 | T | Phosphothreonine:0.062;O-linked_glycosylation:0.866 |
| sp\|P46531\| | 116 | T | Phosphothreonine:0.093;O-linked_glycosylation:0.893 |
| sp\|P46531\| | 131 | P | Hydroxyproline:0.815 |
| sp\|P46531\| | 143 | P | Hydroxyproline:0.578 |
| sp\|P46531\| | 146 | S | Phosphoserine:0.078;O-linked_glycosylation:0.892 |
| sp\|P46531\| | 148 | P | Hydroxyproline:0.809 |
| sp\|P46531\| | 157 | P | Hydroxyproline:0.699 |
| sp\|P46531\| | 168 | P | Hydroxyproline:0.679 |
| sp\|P46531\| | 186 | P | Hydroxyproline:0.511 |
| sp\|P46531\| | 194 | T | Phosphothreonine:0.086;O-linked_glycosylation:0.895 |
| sp\|P46531\| | 213 | P | Hydroxyproline:0.585 |
| sp\|P46531\| | 218 | P | Hydroxyproline:0.62 |
| sp\|P46531\| | 221 | P | Hydroxyproline:0.621 |
| sp\|P46531\| | 223 | S | Phosphoserine:0.7;O-linked_glycosylation:0.179 |
| sp\|P46531\| | 224 | P | Hydroxyproline:0.594 |
| sp\|P46531\| | 225 | S | Phosphoserine:0.633;O-linked_glycosylation:0.196 |
| sp\|P46531\| | 226 | P | Hydroxyproline:0.73 |
| sp\|P46531\| | 232 | T | Phosphothreonine:0.077;O-linked_glycosylation:0.899 |
| sp\|P46531\| | 235 | P | Hydroxyproline:0.706 |
| sp\|P46531\| | 250 | T | Phosphothreonine:0.06;O-linked_glycosylation:0.661 |
| sp\|P46531\| | 262 | P | Hydroxyproline:0.715 |
| sp\|P46531\| | 311 | T | Phosphothreonine:0.069;O-linked_glycosylation:0.909 |
| sp\|P46531\| | 328 | T | Phosphothreonine:0.039;O-linked_glycosylation:0.525 |
| sp\|P46531\| | 341 | S | Phosphoserine:0.072;O-linked_glycosylation:0.906 |
| sp\|P46531\| | 349 | T | Phosphothreonine:0.056;O-linked_glycosylation:0.899 |
| sp\|P46531\| | 378 | S | Phosphoserine:0.064;O-linked_glycosylation:0.889 |
| sp\|P46531\| | 380 | P | Hydroxyproline:0.635 |
| sp\|P46531\| | 391 | P | Hydroxyproline:0.502 |
| sp\|P46531\| | 435 | S | Phosphoserine:0.125;O-linked_glycosylation:0.894 |
| sp\|P46531\| | 445 | T | Phosphothreonine:0.047;O-linked_glycosylation:0.832 |
| sp\|P46531\| | 458 | S | Phosphoserine:0.074;O-linked_glycosylation:0.926 |
| sp\|P46531\| | 466 | T | Phosphothreonine:0.057;O-linked_glycosylation:0.894 |
| sp\|P46531\| | 480 | P | Hydroxyproline:0.609 |
| sp\|P46531\| | 496 | S | Phosphoserine:0.117;O-linked_glycosylation:0.909 |
| sp\|P46531\| | 521 | T | Phosphothreonine:0.043;O-linked_glycosylation:0.598 |
| sp\|P46531\| | 534 | S | Phosphoserine:0.088;O-linked_glycosylation:0.906 |
| sp\|P46531\| | 536 | P | Hydroxyproline:0.607 |
| sp\|P46531\| | 547 | P | Hydroxyproline:0.634 |
| sp\|P46531\| | 559 | T | Phosphothreonine:0.041;O-linked_glycosylation:0.613 |
| sp\|P46531\| | 579 | S | Phosphoserine:0.068;O-linked_glycosylation:0.576 |
| sp\|P46531\| | 593 | P | Hydroxyproline:0.786 |
| sp\|P46531\| | 596 | T | Phosphothreonine:0.051;O-linked_glycosylation:0.675 |
| sp\|P46531\| | 609 | S | Phosphoserine:0.088;O-linked_glycosylation:0.907 |
| sp\|P46531\| | 611 | P | Hydroxyproline:0.591 |
| sp\|P46531\| | 617 | T | Phosphothreonine:0.085;O-linked_glycosylation:0.909 |
| sp\|P46531\| | 634 | T | Phosphothreonine:0.06;O-linked_glycosylation:0.585 |
| sp\|P46531\| | 647 | S | Phosphoserine:0.209;O-linked_glycosylation:0.897 |
| sp\|P46531\| | 648 | S | Phosphoserine:0.534;O-linked_glycosylation:0.279 |
| sp\|P46531\| | 649 | P | Hydroxyproline:0.628 |
| sp\|P46531\| | 652 | S | Phosphoserine:0.133;O-linked_glycosylation:0.805 |
| sp\|P46531\| | 668 | P | Hydroxyproline:0.65 |
| sp\|P46531\| | 671 | T | Phosphothreonine:0.051;O-linked_glycosylation:0.585 |
| sp\|P46531\| | 686 | P | Hydroxyproline:0.787 |
| sp\|P46531\| | 692 | T | Phosphothreonine:0.069;O-linked_glycosylation:0.911 |
| sp\|P46531\| | 722 | S | Phosphoserine:0.126;O-linked_glycosylation:0.901 |
| sp\|P46531\| | 743 | P | Hydroxyproline:0.739 |
| sp\|P46531\| | 746 | S | Phosphoserine:0.08;O-linked_glycosylation:0.709 |
| sp\|P46531\| | 759 | S | Phosphoserine:0.089;O-linked_glycosylation:0.918 |
| sp\|P46531\| | 761 | P | Hydroxyproline:0.781 |
| sp\|P46531\| | 767 | T | Phosphothreonine:0.065;O-linked_glycosylation:0.909 |
| sp\|P46531\| | 784 | S | Phosphoserine:0.077;O-linked_glycosylation:0.892 |
| sp\|P46531\| | 797 | S | Phosphoserine:0.063;O-linked_glycosylation:0.92 |
| sp\|P46531\| | 805 | T | Phosphothreonine:0.067;O-linked_glycosylation:0.912 |
| sp\|P46531\| | 835 | P | Hydroxyproline:0.559 |
| sp\|P46531\| | 836 | S | Phosphoserine:0.636;O-linked_glycosylation:0.183 |
| sp\|P46531\| | 837 | P | Hydroxyproline:0.81 |
| sp\|P46531\| | 876 | S | Phosphoserine:0.544;O-linked_glycosylation:0.175 |
| sp\|P46531\| | 877 | P | Hydroxyproline:0.542 |
| sp\|P46531\| | 883 | S | Phosphoserine:0.085;O-linked_glycosylation:0.861 |
| sp\|P46531\| | 900 | S | Phosphoserine:0.094;O-linked_glycosylation:0.771 |
| sp\|P46531\| | 913 | P | Hydroxyproline:0.707 |
| sp\|P46531\| | 915 | P | Hydroxyproline:0.755 |
| sp\|P46531\| | 921 | S | Phosphoserine:0.068;O-linked_glycosylation:0.907 |
| sp\|P46531\| | 951 | S | Phosphoserine:0.16;O-linked_glycosylation:0.9 |
| sp\|P46531\| | 953 | P | Hydroxyproline:0.798 |
| sp\|P46531\| | 959 | N | N-linked_glycosylation:0.916 |
| sp\|P46531\| | 972 | P | Hydroxyproline:0.627 |
| sp\|P46531\| | 985 | P | Hydroxyproline:0.658 |
| sp\|P46531\| | 997 | T | Phosphothreonine:0.051;O-linked_glycosylation:0.911 |
| sp\|P46531\| | 1011 | P | Hydroxyproline:0.8 |
| sp\|P46531\| | 1014 | T | Phosphothreonine:0.071;O-linked_glycosylation:0.689 |
| sp\|P46531\| | 1027 | S | Phosphoserine:0.081;O-linked_glycosylation:0.894 |
| sp\|P46531\| | 1029 | P | Hydroxyproline:0.751 |
| sp\|P46531\| | 1035 | T | Phosphothreonine:0.064;O-linked_glycosylation:0.897 |
| sp\|P46531\| | 1048 | P | Hydroxyproline:0.533 |
| sp\|P46531\| | 1065 | S | Phosphoserine:0.077;O-linked_glycosylation:0.902 |
| sp\|P46531\| | 1067 | P | Hydroxyproline:0.751 |
| sp\|P46531\| | 1138 | T | Phosphothreonine:0.07;O-linked_glycosylation:0.502 |
| sp\|P46531\| | 1153 | P | Hydroxyproline:0.699 |
| sp\|P46531\| | 1159 | T | Phosphothreonine:0.07;O-linked_glycosylation:0.904 |
| sp\|P46531\| | 1179 | N | N-linked_glycosylation:0.913 |
| sp\|P46531\| | 1189 | S | Phosphoserine:0.076;O-linked_glycosylation:0.902 |
| sp\|P46531\| | 1197 | T | Phosphothreonine:0.08;O-linked_glycosylation:0.909 |
| sp\|P46531\| | 1202 | P | Hydroxyproline:0.676 |
| sp\|P46531\| | 1235 | S | Phosphoserine:0.788;O-linked_glycosylation:0.482 |
| sp\|P46531\| | 1241 | N | N-linked_glycosylation:0.917 |
| sp\|P46531\| | 1243 | T | Phosphothreonine:0.077;O-linked_glycosylation:0.845 |
| sp\|P46531\| | 1256 | P | Hydroxyproline:0.51 |
| sp\|P46531\| | 1257 | P | Hydroxyproline:0.777 |
| sp\|P46531\| | 1273 | S | Phosphoserine:0.152;O-linked_glycosylation:0.901 |
| sp\|P46531\| | 1281 | T | Phosphothreonine:0.094;O-linked_glycosylation:0.756 |
| sp\|P46531\| | 1306 | S | Phosphoserine:0.524;O-linked_glycosylation:0.095 |
| sp\|P46531\| | 1315 | P | Hydroxyproline:0.858 |
| sp\|P46531\| | 1321 | T | Phosphothreonine:0.066;O-linked_glycosylation:0.858 |
| sp\|P46531\| | 1354 | S | Phosphoserine:0.344;O-linked_glycosylation:0.766 |
| sp\|P46531\| | 1362 | T | Phosphothreonine:0.089;O-linked_glycosylation:0.91 |
| sp\|P46531\| | 1367 | P | Hydroxyproline:0.682 |
| sp\|P46531\| | 1369 | S | Phosphoserine:0.629;O-linked_glycosylation:0.178 |
| sp\|P46531\| | 1370 | P | Hydroxyproline:0.599 |
| sp\|P46531\| | 1379 | T | Phosphothreonine:0.07;O-linked_glycosylation:0.86 |
| sp\|P46531\| | 1388 | S | Phosphoserine:0.07;O-linked_glycosylation:0.581 |
| sp\|P46531\| | 1390 | P | Hydroxyproline:0.551 |
| sp\|P46531\| | 1396 | P | Hydroxyproline:0.553 |
| sp\|P46531\| | 1402 | T | Phosphothreonine:0.073;O-linked_glycosylation:0.886 |
| sp\|P46531\| | 1489 | N | N-linked_glycosylation:0.921 |
| sp\|P46531\| | 1532 | Y | Phosphotyrosine:0.584 |
| sp\|P46531\| | 1584 | Q | Pyrrolidone_carboxylic_acid:0.653 |
| sp\|P46531\| | 1587 | N | N-linked_glycosylation:0.894 |
| sp\|P46531\| | 1589 | S | Phosphoserine:0.568;O-linked_glycosylation:0.07 |
| sp\|P46531\| | 1657 | S | Phosphoserine:0.643;O-linked_glycosylation:0.101 |
| sp\|P46531\| | 1674 | S | Phosphoserine:0.551;O-linked_glycosylation:0.07 |
| sp\|P46531\| | 1677 | Y | Phosphotyrosine:0.676 |
| sp\|P46531\| | 1723 | S | Phosphoserine:0.1;O-linked_glycosylation:0.54 |
| sp\|P46531\| | 1725 | T | Phosphothreonine:0.134;O-linked_glycosylation:0.872 |
| sp\|P46531\| | 1759 | K | Ubiquitination:0.862;SUMOylation:0.034;N6-acetyllysine:0.13;Methyllysine:0.032 |
| sp\|P46531\| | 1763 | Q | Pyrrolidone_carboxylic_acid:0.891 |
| sp\|P46531\| | 1766 | Q | Pyrrolidone_carboxylic_acid:0.61 |
| sp\|P46531\| | 1791 | S | Phosphoserine:0.766;O-linked_glycosylation:0.058 |
| sp\|P46531\| | 1795 | K | Ubiquitination:0.221;SUMOylation:0.048;N6-acetyllysine:0.663;Methyllysine:0.10 |
| sp\|P46531\| | 1799 | N | N-linked_glycosylation:0.859 |
| sp\|P46531\| | 1801 | S | Phosphoserine:0.828;O-linked_glycosylation:0.085 |
| sp\|P46531\| | 1861 | T | Phosphothreonine:0.831;O-linked_glycosylation:0.401 |
| sp\|P46531\| | 1900 | S | Phosphoserine:0.808;O-linked_glycosylation:0.161 |
| sp\|P46531\| | 1922 | N | N-linked_glycosylation:0.902 |
| sp\|P46531\| | 1951 | S | Phosphoserine:0.709;O-linked_glycosylation:0.112 |
| sp\|P46531\| | 2029 | K | Ubiquitination:0.587;SUMOylation:0.055;N6-acetyllysine:0.124;Methyllysine:0.07 |
| sp\|P46531\| | 2063 | T | Phosphothreonine:0.616;O-linked_glycosylation:0.098 |
| sp\|P46531\| | 2121 | S | Phosphoserine:0.834;O-linked_glycosylation:0.069 |
| sp\|P46531\| | 2132 | T | Phosphothreonine:0.721;O-linked_glycosylation:0.304 |
| sp\|P46531\| | 2134 | T | Phosphothreonine:0.292;O-linked_glycosylation:0.688 |
| sp\|P46531\| | 2136 | S | Phosphoserine:0.757;O-linked_glycosylation:0.15 |
| sp\|P46531\| | 2141 | S | Phosphoserine:0.737;O-linked_glycosylation:0.107 |
| sp\|P46531\| | 2162 | S | Phosphoserine:0.671;O-linked_glycosylation:0.078 |
| sp\|P46531\| | 2170 | S | Phosphoserine:0.512;O-linked_glycosylation:0.048 |
| sp\|P46531\| | 2183 | S | Phosphoserine:0.714;O-linked_glycosylation:0.044 |
| sp\|P46531\| | 2198 | S | Phosphoserine:0.781;O-linked_glycosylation:0.064 |
| sp\|P46531\| | 2202 | S | Phosphoserine:0.573;O-linked_glycosylation:0.073 |
| sp\|P46531\| | 2205 | S | Phosphoserine:0.646;O-linked_glycosylation:0.078 |
| sp\|P46531\| | 2215 | S | Phosphoserine:0.733;O-linked_glycosylation:0.216 |
| sp\|P46531\| | 2221 | S | Phosphoserine:0.571;O-linked_glycosylation:0.402 |
| sp\|P46531\| | 2226 | S | Phosphoserine:0.642;O-linked_glycosylation:0.432 |
| sp\|P46531\| | 2252 | K | Ubiquitination:0.247;SUMOylation:0.661;N6-acetyllysine:0.227;Methyllysine:0.08 |
| sp\|P46531\| | 2263 | R | Methylarginine:0.594 |
| sp\|P46531\| | 2272 | R | Methylarginine:0.57 |
| sp\|P46531\| | 2295 | N | N-linked_glycosylation:0.919 |
| sp\|P46531\| | 2316 | S | Phosphoserine:0.561;O-linked_glycosylation:0.087 |
| sp\|P46531\| | 2327 | R | Methylarginine:0.772 |
| sp\|P46531\| | 2337 | T | Phosphothreonine:0.113;O-linked_glycosylation:0.615 |
| sp\|P46531\| | 2361 | Q | Pyrrolidone_carboxylic_acid:0.559 |

| sp\|P46531\| | 2372 | R | Methylarginine:0.855 |
| --- | --- | --- | --- |
| sp\|P46531\| | 2432 | S | Phosphoserine:0.622;O-linked_glycosylation:0.058 |
| sp\|P46531\| | 2435 | S | Phosphoserine:0.564;O-linked_glycosylation:0.116 |
| sp\|P46531\| | 2466 | T | Phosphothreonine:0.097;O-linked_glycosylation:0.658 |
| sp\|P46531\| | 2477 | T | Phosphothreonine:0.068;O-linked_glycosylation:0.527 |
| sp\|P46531\| | 2480 | Q | Pyrrolidone_carboxylic_acid:0.505 |
| sp\|P46531\| | 2483 | T | Phosphothreonine:0.462;O-linked_glycosylation:0.512 |
| sp\|P46531\| | 2513 | S | Phosphoserine:0.608;O-linked_glycosylation:0.104 |
| sp\|P46531\| | 2516 | S | Phosphoserine:0.649;O-linked_glycosylation:0.073 |
| sp\|P46531\| | 2528 | N | N-linked_glycosylation:0.865 |
| sp\|P46531\| | 2538 | S | Phosphoserine:0.731;O-linked_glycosylation:0.126 |
