# Supplementary material for: Neurogenic locus notch homolog protein 1 (NOTCH 1) SNP informatics coupled with intrinsically disordered regions and post-translational modifications reveals the complex structural crosstalk of Lung Adenocarcinoma (LUAD)
Source: Front Bioinform. 2025 Dec 10;5:1641521. doi: 10.3389/fbinf.2025.1641521 (PMC12727990; doi:10.3389/fbinf.2025.1641521)
Supplement: Supplementary file 5 [file Table3.docx]

**Supplementary Table 3: Estimated Molecular Docking measures of HADDOCK for WILDTYPE, Mutant1 (S1464I) Mutant 2 (T1602I) and Mutant 3 (A1705V)**

| **Wild type NOTCH1 Docking with the interactors** | | | | | | | | | | | | |
| --- | --- | --- | --- | --- | --- | --- | --- | --- | --- | --- | --- | --- |
| Interactors | Haddock Score | Cluster Size | | RMSD | | VanderWaals Energy | Electrostatic Energy | | Desolvation Energy | Restraints violation energy | Buried Surface Area | Z-Score |
| MAML1 | -62.5+/-3.9 | 8 | | 16.9+/-0.6 | | -33.4+/-3.2 | -142.9+/-20.2 | | 0.5+/-2.8 | 0.0+/-0.0 | 1233.2+/-37.7 | -1.8 |
| PSEN1 | -52.5+/-3.9 | 23 | | 33.0+/-0.7 | | -15.7+/-2.8 | -119.2+/-17.9 | | 13.0+/-3.6 | 0.0+/-0.0 | 661.4+/-110.9 | -1.8 |
| MAML2 | -64.8+/-8.6 | 4 | | 12.9+/-0.2 | | -46.2+/-6.2 | -114.0+/-16.5 | | 3.2+/-2.5 | 10.5+/-17.8 | 1600.9+/-288.4 | -1.3 |
| JAG2 | -65.2+/-7.1 | 6 | | 22.8+/-0.2 | | -42.9+/-10.7 | -96.3+/-23.3 | | -3.2+/-0.7 | 2.1+/-1.2 | 1290.7+/-160.9 | -1.3 |
| JAG1 | -70.4+/-2.5 | 28 | | 10.0+/-0.3 | | -18.2+/-2.5 | -209.6+/-10.8 | | -10.3+/-1.6 | 1.6+/-0.9 | 1014.7+/-97.6 | -1.7 |
| DLL4 | -62.3+/-3.2 | 26 | | 21.3+/-0.2 | | -37.0+/-3.0 | -134.8+/-10.5 | | 1.7+/-1.1 | 0.0+/-0.0 | 1233.1+/-32.4 | -1.5 |
| **Mutant S1464I Docking with the interactors** | | | | | | | | | | | | |
| Interactors | Haddock Score | Cluster Size | | RMSD | | VanderWaals Energy | Electrostatic Energy | Desolvation Energy | | Restraints violation energy | Buried Surface Area | Z-Score |
| MAML1 | -59.7+/-9.1 | 7 | | 18.2+/-0.2 | | 28.5+/-5.6 | -165.7+/-30.4 | 1.9+/-0.9 | | 0.1+/-0.1 | 1044.3+/-55.7 | -1.9 |
| PSEN1 | -55.4+/-1.9 | 9 | | 16.5+/-0.5 | | -26.8+/-1.6 | -99.5+/-8.3 | -8.6+/-4.1 | | 0.0+/-0.0 | 973+/-77.6 | -2.0 |
| MAML2 | -63.8+/-6.7 | 4 | | 2.0+/-0.3 | | -28.9+/-4.9 | -212.1+/-24.1 | 4.5+/-2.7 | | 29.1+/-16.9 | 1491.3+/-105.4 | -1.4 |
| JAG2 | -56.4+/-8.1 | 5 | | 21.9+/-0.2 | | -32.0+/-5.2 | -127.8+/-29.9 | -2.1+/-2.4 | | 32.7+/-3.8 | 1481.5+/-134.9 | -1.5 |
| JAG1 | -73.2+/-7.9 | 11 | | 26.0+/-0.1 | | -18.4+/-7.5 | -197.7+/-15.9 | 15.3+/-2.4 | | 0.7+/-0.6 | 983.7+/-91.6 | -1.4 |
| DLL4 | -64.3+/-1.9 | 33 | | 20.0+/-0.2 | | -37.8+/-2.8 | -132.7+/-13.6 | 0.1+/-3.2 | | 0.0+/-0.0 | 1252.3+/-49.3 | -1.7 |
| **Mutant T1602I Docking with the interactors** | | | | | | | | | | | | |
| Interactors | Haddock Score | Cluster Size | | RMSD | | VanderWaals Energy | Electrostatic Energy | Desolvation Energy | | Restraints violation energy | Buried Surface Area | Z-Score |
| MAML1 | -64.6+/-8.6 | 10 | | 17.1+/-0.2 | | -35.7+/-4.1 | -134.7+/-40.2 | -1.9+/-1.8 | | 0.1+/-0.1 | 1139.0+/-83.2 | -1.7 |
| PSEN1 | -53.1+/-4.9 | 9 | | 30.0+/-0.3 | | -28.5+/-3.1 | -76.0+/-16.6 | -9.5+/-5.0 | | 0.8+/-1.4 | 997.1+/-76.8 | -1.4 |
| MAML2 | -64.6+/-5.4 | 18 | | 1.5+/-0.9 | | -43.8+/-4.2 | -131.3+/-20.2 | 5.4+/-2.7 | | 0.1+/-0.1 | 1713.3+/-167.4 | -1.2 |
| JAG2 | -79.9+/-12.7 | 5 | | 1.6+/-1.2 | | 41.6+/-8.1 | 154.4+/-8.0 | -9.5+/-2.9 | | 20.6+/-13.5 | 1339.2+/-169.7 | -1.7 |
| JAG1 | -75.9+/-4.5 | 31 | | 2.0+/-0.6 | | 19.9+/-1.8 | -232.4+/-24.9 | -9.5+/-0.6 | | 0.6+/-0.4 | 1212.6+/-49.5 | -1.6 |
| DLL4 | -61.8+/-7.6 | 5 | | 28.0+/-0.3 | | -33.1+/-4.7 | -123.2+/-25.1 | -4.2+/-1.2 | | 0.9+/-1.6 | 1191.4+/-98.5 | -1.9 |
| **Mutant A1705V Docking with the interactors** | | | | | | | | | | | | |
| Interactors | Haddock Score | | Cluster Size | | RMSD | VanderWaals Energy | Electrostatic Energy | | Desolvation Energy | Restraints violation energy | Buried Surface Area | Z-Score |
| MAML1 | -62.7+/-1.3 | | 10 | | 19.5+/-0.4 | -37.5+/-2.3 | -111.5+/-12.3 | | -3.0+/-2.5 | 0.6+/-0.6 | 1152.0+/-25.8 | -1.4 |
| PSEN1 | -55.4+/-3.1 | | 29 | | 30.4+/-0.7 | -23.1+/-3.2 | 74.2+/-22.9 | | -17.5+/-3.5 | 0.0+/-0.0 | 762.3+/-62.7 | -1.4 |
| MAML2 | -69.8+/-9.9 | | 4 | | 11.1+/-0.2 | -46.8+/-6.6 | 117.3+/-25.5 | | 0.5+/-3.9 | 0.1+/-0.1 | 1649.3+/-240.3 | -1.3 |
| JAG2 | -79.0+/-7.4 | | 6 | | 21.1+/-0.1 | -45.0+/-5.3 | -1538+/-34.8 | | -3.4+/-1.6 | 1.2+/-0.3 | 1403.6+/-122.0 | -1.5 |
| JAG1 | -68.7+/-1.5 | | 22 | | 19.7+/-0.2 | -14.0+/-2.6 | -233.3+/-22.2 | | -8.1+/-1.3 | 1.0+/-0.7 | 1057.4+/-59.4 | -1.5 |
| DLL4 | -63.1+/-3.0 | | 28 | | 24.8+/-0.3 | 43.1+/-0.7 | -88.3+/-13.5 | | -2.4+/-0.8 | 0.5+/-0.8 | 1235.2+/-32.0 | -1.5 |
